# Prognostic and predictive significance of circulating biomarkers in patients with advanced upper gastrointestinal cancer undergoing systemic chemotherapy

**DOI:** 10.3389/fonc.2023.1195848

**Published:** 2023-06-06

**Authors:** Ningning Li, Liwei Gao, Yuping Ge, Lin Zhao, Chunmei Bai, Yingyi Wang

**Affiliations:** ^1^ Department of Medical Oncology, Peking Union Medical College Hospital, Chinese Academy of Medical Sciences and Peking Union Medical College, Beijing, China; ^2^ Department of Radiation Oncology, China-Japan Friendship Hospital, Beijing, China

**Keywords:** esophageal cancer, gastric cancer, chemotherapy, prognosis, biomarker

## Abstract

**Objective:**

The prognosis of patients with advanced cancers of the upper gastrointestinal (UGI) tract is poor. Systemic chemotherapy forms the basis for their treatment, with limited efficacy. Biomarkers have been introduced into clinical practice for cancer management. This study aimed to investigate the predictive and prognostic values of circulating biomarkers in patients with advanced esophageal and gastric cancers receiving chemotherapy.

**Design:**

Overall, 92 patients with advanced esophageal squamous cell carcinoma (ESCC; *n* = 38) and gastric adenocarcinoma (GAC; *n* = 54) were enrolled. We analyzed the association of circulating lymphocyte subsets, inflammatory markers, and blood cell counts with treatment efficacy and patient survival.

**Results:**

Significant differences were identified in peripheral blood parameters between the groups with different clinicopathological features. Hemoglobin (Hb, *p* = 0.014), eosinophil counts (*p* = 0.028), CD4^+^CD28^+^T/CD4^+^T percentage (*p* = 0.049), CD8^+^CD38^+^T/CD8^+^T percentage (*p* = 0.044), memory CD4^+^T (*p* = 0.007), and CD4^+^CD28^+^T (*p* = 0.007) were determined as predictors for achieving non-PD (progression disease) in the ESCC cohort. High levels of eosinophils (*p* = 0.030) and memory CD4^+^T cells (*p* = 0.026) and high eosinophil-to-lymphocyte ratio (ELR, *p* = 0.013) were predictors of non-PD in patients with GAC. The combined detection models exhibited good ability to distinguish between partial response (PR)/non-PR and PD/non-PD in patients with ESCC and GAC, respectively. Using the multivariate Cox model, the Eastern Cooperative Oncology Group (ECOG) score status (hazard ratio [HR]: 4.818, 95% confidence intervals [CI]: 2.076–11.184, *p* < 0.001) and eosinophil count (HR: 0.276, 95% CI: 0.120–0.636, *p* = 0.003) were independent prognostic factors of progression-free survival (PFS) in patients with ESCC. Metastatic sites (HR: 2.092, 95% CI: 1.307–3.351, *p* = 0.002) and eosinophil-to-lymphocyte ratio (ELR; HR: 0.379, 95% CI: 0.161–0.893, *p* = 0.027) were independent prognostic factors for overall survival (OS) in patients with ESCC. Differentiation (HR: 0.041, 95% CI: 0.200–0.803, *p* = 0.010), memory CD4^+^T (HR: 0.304, 95% CI: 0.137–0.675, *p* = 0.003), NK cells (HR: 2.302, 95% CI: 1.044–3.953, *p* = 0.037), and C-reactive protein-to-lymphocyte ratio (CLR; HR: 2.070, 95% CI: 1.024–4.186, *p* = 0.043) were independent prognostic factors for PFS in patients with GAC. Total lymphocyte counts (HR: 0.260, 95% CI: 0.086–0.783, *p* = 0.017), CD8^+^T (HR: 0.405, 95% CI: 0.165–0.997, *p* = 0.049), NK cells (HR: 3.395, 95% CI: 1.592–7.238, *p* = 0.002), and monocyte-to-lymphocyte ratio (MLR; HR: 3.076, 95% CI: 1.488–6.360, *p* = 0.002) were identified as independent prognostic factors associated with OS of GAC.

**Conclusion:**

Lymphocyte subsets, blood cell counts, and inflammatory parameters may predict the chemotherapeutic response and prognosis in ESCC and GAC. A combination of these markers can be used to stratify patients into risk groups, which could improve treatment strategies.

## 1 Introduction

In 2020, 19.3 million new cases of cancer were diagnosed worldwide, and the number of cancer-related deaths was estimated to be 10.0 million (1). Primary malignant tumors of the upper gastrointestinal (UGI) tract account for a large proportion of cancer cases, particularly in Asian countries. Tobacco and alcohol exposure has a combined carcinogenic effect on UGI (2). Esophageal cancer (EC) was the sixth most common cause of cancer-related deaths in China in 2020 (3). Esophageal squamous cell carcinoma (ESCC) is more common in Asian countries, while adenocarcinoma (AC) is more common in Western countries (4). Although new techniques and drug regimens have been developed, treating patients with advanced or metastatic EC remains extremely difficult. Gastric cancer (GC) is the third most common cancer in China, causing 374,000 deaths in 2020 (3). Recent research on immune checkpoint inhibitors has shown that they can be a promising strategy for treating advanced EC and GC; however, the prognosis remains unfavorable.

To date, several studies have investigated peripheral blood parameters to identify biomarkers for predicting treatment efficacy or prognosis and help clinicians develop better therapeutic strategies. These markers are associated with various factors. Systemic inflammation and nutrition are both associated with carcinogenesis and cancer progression (5–7). Common biochemical inflammatory markers include C-reactive protein (CRP) level, platelet-to-lymphocyte ratio (PLR), monocyte-to-lymphocyte ratio (MLR), and neutrophil-to-lymphocyte ratio (NLR) (8–10). Albumin and prealbumin levels are indicators of nutritional status and are associated with poor prognosis (11). Additionally, the CRP/albumin ratio (CAR), a novel combined biomarker, has demonstrated prognostic significance in EC and GC (12, 13). Several studies have indicated that immune responses and the tumor microenvironment are key in cancer progression and prognosis (14). Lymphocyte subsets are markers that reflect the immune response in the tumor microenvironment (15). A previous study revealed that high circulating lymphocyte ratios before and after neoadjuvant chemotherapy were positively associated with pathological complete response (pCR) and improved overall survival (OS) in advanced GC (16). Chemoradiotherapy-induced increase in CD4^+^T cell ratios can predict superior progression-free survival (PFS), while increased CD8^+^T cell ratios can predict improved OS in ESCC (17). Based on the inflammatory and immune-modulating effects on cancer, combining inflammation-related and immune parameters to predict efficacy and prognosis can be significant.

Therefore, the aim of this study was to explore the correlation of blood cell counts, inflammatory markers, lymphocyte subsets, and some combined parameters with the therapeutic response and clinical outcomes in patients with UGI cancer. We conducted a prospective study to determine the clinical significance of these serum markers and fecal microbiota to help guide individualized treatment for patients with UGI cancer.

## 2 Materials and methods

### 2.1 Study population and treatment protocol

A total of 92 patients with UGI cancer, admitted between 2018 and 2020 at Peking Union Medical College Hospital (PUMCH), were enrolled in this study. The recruited patients were pathologically confirmed to have ESCC or gastric adenocarcinoma (GAC). Eligible patients were confirmed to have locally advanced or distant metastatic disease according to the American Joint Committee on Cancer’s (AJCC) Eighth Staging Manual.

Patients in both the ESCC and GAC cohorts received chemotherapy according to the physician’s choice. All patients received systemic chemotherapy every 3 weeks. Chemotherapy was performed mostly using the TP regimen (paclitaxel 175 mg/m^2^, day 1; cisplatin 25 mg/m^2^, days 1–3) in the ESCC cohort. SOX/XELOX (oxaliplatin 130 mg/m^2^, day 1; S-1 40–60 mg bid or capecitabine 1 g/m^2^ bid, days 1–14) regimen was commonly administered to the GAC cohort. Clinical efficacy was assessed 6 weeks after chemotherapy according to the Response Evaluation Criteria in Solid Tumors (RECIST, v1.1). All the participants provided written informed consent to participate in the study. All procedures were performed in accordance with ethical standards, and this study was approved by the Ethics Committee of PUMCH.

The clinical data of the participants were collected from their medical records. Whole blood cell counts; albumin, prealbumin, and lactate dehydrogenase (LDH) levels; lymphocyte subsets; erythrocyte sedimentation rate (ESR); and hypersensitive C-reactive protein (CRP) levels were measured in all patients in the week before chemotherapy. Combined parameters, like the monocyte-to-lymphocyte ratio (MLR), neutrophil-to-lymphocyte ratio (NLR), eosinophil-to-lymphocyte ratio (ELR), basophil-to-lymphocyte ratio (BLR), platelet-to-lymphocyte ratio (PLR), CRP-to-albumin ratio (CAR), CRP-to-prealbumin ratio (COP), CRP-to-lymphocyte ratio (CLR), and CRP-to-body mass index (BMI) ratio (CBR), were calculated and analyzed.

### 2.2 Flow cytometry and blood tests

Three-color flow cytometry (Epics XL flow cytometry; Beckman Coulter, USA) was performed to determine the lymphocyte subsets. Cells were stained with fluorescein-labeled specific monoclonal antibodies against CD4, CD8, CD45RO, CD45RA, CD19, CD16, CD56, CD28, HLA-DR, and CD38. Absolute blood cell counts were determined using an automated cell counter (SYSMEX, XN9100) as part of routine blood tests.

### 2.3 Follow-up

The patients were followed up every 6–8 weeks during treatment, every 3 months after the 6-month chemotherapy course during the first 2 years, and then annually until death. The last follow-up date was 1 March 2022.

### 2.4 Statistical analysis

Data processing and statistical analyses were performed using SPSS 26.0 (IBM Corp., Armonk, NY, USA). Comparisons between groups were performed using *t*-tests and one-way analysis of variance for parametric data. The Mann–Whitney and Kruskal–Wallis tests were used for nonparametric data. Receiver operating characteristic (ROC) curve analysis was performed to evaluate the predictive ability of biomarkers for differentiating patients with different treatment responses. The cutoff values were estimated at various sensitivities and specificities and were determined using the maximized Youden’s index (Sensitivity + Specificity − 1). Kaplan–Meier analysis was performed, and the log-rank test was used to compare PFS and OS. Cox regression analysis was used for univariate and multivariate analyses to assess independent prognostic factors. All statistical tests were two-sided, and *p* < 0.05 was considered statistically significant. GraphPad Prism 8.0 software (version 8.3.1, USA) was used to conduct statistical analyses and prepare figures.

## 3 Results

### 3.1 Patient characteristics

The ESCC and GAC cohorts comprised 38 and 54 patients, respectively. The demographic and clinical characteristics of the participants are summarized in **Table 1**. Participants were divided into three age groups according to the World Health Organization classification (0–44 years, 45–59 years, and >59 years). Both the ESCC and GAC cohorts showed a male predominance. A total of 45 patients were identified as having locally advanced-stage cancer, while 47 had distant metastases. Chemotherapy mainly comprised two standard clinical regimens. Blood cell counts, inflammatory parameters, and lymphocyte subsets in both cohorts are presented in **Table S1**. The calculated combined parameters are listed in **Table S2**.

**Table 1:** Characteristics of UGI cancer patients.

Features	Esophageal cancer (n= 38)	Gastric cancer (n=54)	Total (n=92)
Age			
Young ( 0-44)	0	8	8
Middle (45-59)	9	12	21
Elderly (>59)	29	34	63
Gender (Male/Female)	32/6	37/17	69/23
Histology			
Adenocarcinoma	0	54	54
Squamous carcinoma	38	0	38
Differentiation			
Poorly	9	35	44
Middle-High	29	19	48
Staging of patients			
Locally advanced	25	20	45
Distant metastases	13	34	47
Metastasis sites			
0	27	21	48
1	3	21	24
>2	8	12	20
ECOG PS			
0-1	20	33	53
2-4	18	21	39
BMI (median )	21.84	22.0	22.0
Anti-tumor regimen			
Oxaliplatin+S-1 /capecitabine	1	48	49
Taxane+cisplatin	37	6	43
Adverse reaction of therapy			
Grade 1-2	29	47	76
Grade 3-4	9	7	16
Response			
PR	9	18	27
SD	21	26	47
PD	8	10	18

ECOG-PS, Eastern Cooperative Oncology Group Performance Status; BMI, Body Mass Index; PR, Partial response; SD, Stable Disease; PD, Progression Disease.

### 3.2 Relationships between circulating biomarkers and clinicopathological features

We compared all peripheral blood markers between the different subgroups using *t*-tests, Mann–Whitney *U* tests, and Kruskal–Wallis tests. There were no statistically significant differences in white blood cell (WBC), eosinophil, or basophil counts between the groups for tumor type (ESCC vs. GAC), differentiation (poor vs. middle-high), staging (locally advanced vs. metastasis), number of metastatic sites, or ECOG score (0–1 vs. 2–4) (**Figure S1**).

**Figure 1:**
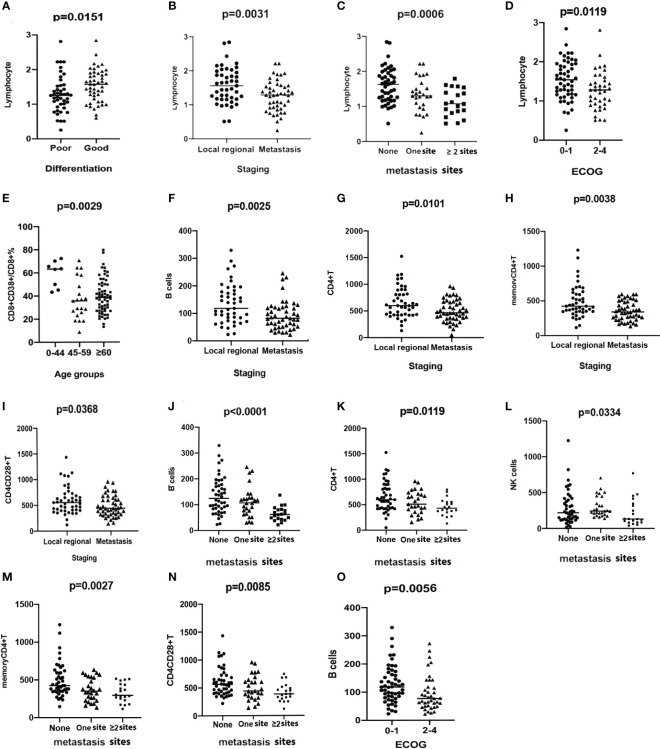
Scatter plots demonstrating the comparison of the counts of lymphocytes between groups stratified by differentiation **(A)**, staging **(B)**, the number of metastasis sites **(C)**, and ECOG status **(D)**. Comparison of the percentage of CD8^+^CD38^+^T/CD8^+^T among groups stratified by ages **(E)**. The levels of B cells **(F)**, CD4^+^T **(G)**, memory CD4^+^T **(H)**, and CD4^+^CD28^+^T cells **(I)** are compared between groups with different stages. The levels of B cells **(J)**, CD4^+^T **(K)**, NK cells **(L)**, memory CD4^+^T **(M)**, and CD4^+^CD28^+^T cells **(N)** are compared among groups stratified by the number of metastatic sites. B cells **(O)** are compared and demonstrated in scatter plots between groups with different ECOG status.

However, statistically significant differences were observed in several parameters. Higher levels of platelets (PLT) were observed in the ECOG 2–4 group (*p* = 0.0209) than in the ECOG 0–1 group (**Figure S2**). Significantly lower levels of total lymphocytes were observed in the groups with poor differentiation (*p* = 0.0151, **Figure 1A**), distant metastatic disease (*p* = 0.0031, **Figure 1B**), more metastatic sites (*p* = 0.0006, **Figure 1C**), and worse ECOG scores (*p* = 0.0119, **Figure 1D**). Neutrophils were significantly higher in the groups with distant metastatic disease (*p* = 0.0159), more metastatic sites (*p* = 0.0412), and worse ECOG scores (*p* = 0.0096) (**Figure S2**). ESR, hsCRP, and LDH levels showed a significant trend toward higher levels in more advanced cancers and worse ECOG status (*p* < 0.05) (**Figure S2**). Both albumin and prealbumin levels were lower in the groups with more metastatic sites and worse ECOG scores (*p* < 0.05, **Figure S2**).

To explore the reason for individual differences in circulating immune parameters, the relative and absolute numbers of lymphocyte subsets were quantified (**Figure S3**) and divided into groups according to age, tumor type, stage, metastatic site, and ECOG performance status. A significant decrease in the percentage of CD8^+^CD38^+^T/CD8^+^T was noted in the patients in the 45–59-year and ≥60-year groups, when separately compared with those in the 0–44-year group (**Figure 1E**). Significantly higher level of CD8^+^CD28^+^T cells were observed in the 45–59-year group compared with that in the other age groups (**Figure S2**). No significant differences were observed in the other subsets among the age groups.

Higher levels of memory CD4^+^ (*p* = 0.0197), CD4^+^CD28^+^ (*p* = 0.0138), and CD8^+^CD28^+^T cells (*p* = 0.0398) were observed in patients with ESCC than in patients with GAC. However, the percentage of CD8^+^CD38^+^/CD8^+^T was higher in the GAC group (*p* = 0.0056, **Figure S2**), compared with the ESCC group. Other lymphocyte subsets were not significantly different between patients with ESCC and those with GAC. There was a significant decrease in the number of B cells (*p* = 0.0025, **Figure 1F**), CD4^+^T cells (*p* = 0.0101, **Figure 1G**), memory CD4^+^T cells (*p* = 0.0038, **Figure 1H**), and CD4^+^CD28^+^T cells (*p* = 0.0368, **Figure 1I**) in patients with distant metastasis compared to that in patients with locally advanced disease. A clear decreasing trend in the numbers of B cells (*p* < 0.0001, **Figure 1J**), CD4^+^T cells (*p* = 0.0119, **Figure 1K**), NK cells (*p* = 0.0334, **Figure 1L**), memory CD4^+^T cells (*p* = 0.0027, **Figure 1M**), and CD4^+^CD28^+^T cells (*p* = 0.0085, **Figure 1N**) was detected with the worsening of metastasis. B-cell counts were significantly lower in patients with ECOG 2–4, compared with patients with ECOG 0–1 (*p* = 0.0056, **Figure 1O**).

Combined serum indicators based on inflammatory, biochemical, and immune parameters were evaluated in different clinical groups. No differences were observed in any of the nine combined parameters between the ESCC and AGC cohorts. NLR (*p* = 0.0349, **Figure S2**) and PLR (*p* = 0.0201, **Figure S2**) demonstrated higher levels in patients with poorly differentiated tumors than those with well-differentiated ones. All combined parameters were significantly higher in metastatic tumors than in locally advanced ones, except for ELR (**Figure S2**). MLR (*p* = 0.0063), NLR (*p* < 0.0001), PLR (*p* = 0.0001), CAR (*p* = 0.0007), COP (*p* = 0.0004), CLR (*p* < 0.0001), and CBR (*p* = 0.0022), which were significantly higher in patients with a worse ECOG status (**Figure S2**).

### 3.3 Relationships between tumor responses and circulating biomarkers

The efficacy of chemotherapy was assessed in all 92 patients. **Table 1** shows the responses to chemotherapy. Nine patients in the ESCC cohort experienced partial response (PR); the overall response rate (ORR) and disease control rate (DCR) were 23.7% and 78.9%, respectively. Eighteen patients in the GAC cohort experienced PR, with ORR and DCR being 33.3% and 81.5%, respectively.

To explore whether the circulating parameters were associated with efficacy, we performed the Mann–Whitney *U* test to observe the differences between groups (PR and non-PR; non-PD and PD) with chemotherapy outcomes in the ESCC and GAC cohorts, respectively. All the mean ± standard deviation (SD) values for the patients of different subgroups are displayed in **Tables S3**, **S4**.

**Figure 2:**
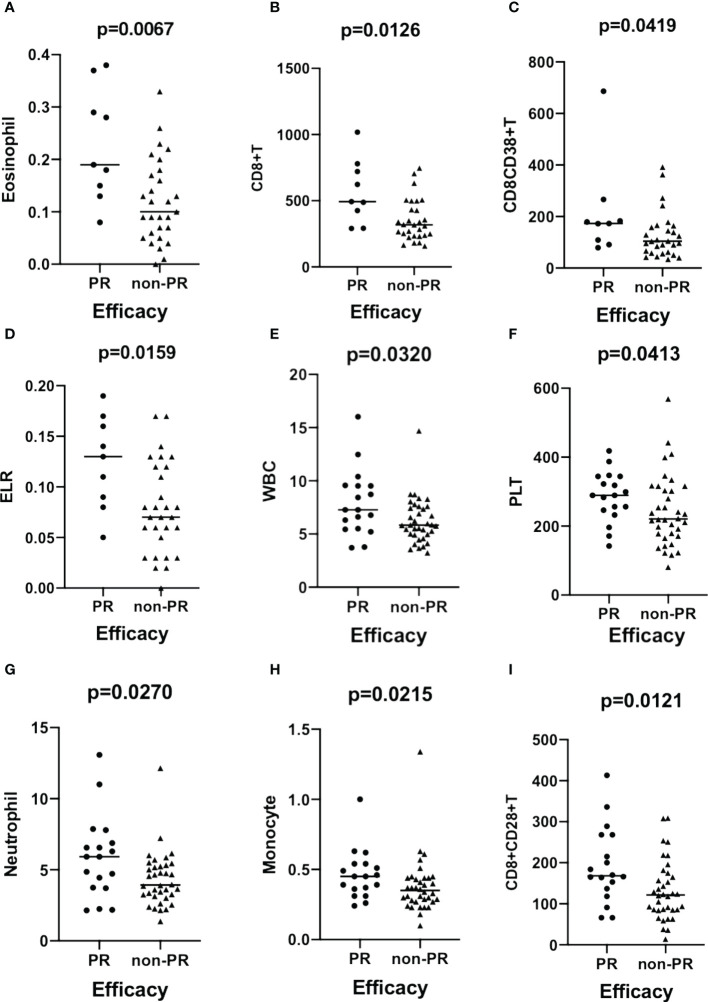
Scatter plots demonstrating the comparison of the counts of eosinophil **(A)**, CD8^+^T **(B)**, CD8^+^CD38^+^T **(C)**, and ELR **(D)** between groups stratified by efficacy (PR vs. non-PR) in patients with ESCC. The levels of WBC **(E)**, PLT **(F)**, neutrophils **(G)**, monocytes **(H)**, and CD8^+^CD28^+^T cells **(I)** are compared between groups with different efficacies (PR vs. non-PR) in patients with GAC. ESCC, esophageal squamous carcinoma; GAC, gastric adenocarcinoma.

The results demonstrated that the eosinophil count (*p* = 0.0067, **Figure 2A**), CD8^+^T cell count (*p* = 0.0126, **Figure 2B**), CD8^+^CD38^+^T cell count (*p* = 0.0419, **Figure 2C**), and ELR (*p* = 0.0159, **Figure 2D**) were significantly higher in patients with PR than in those with non-PR in the ESCC cohort. Moreover, the total lymphocyte count (*p* = 0.0420; **Figure S4**), levels of prealbumin (*p* = 0.0039, **Figure S4**) and albumin (*p* = 0.0087, **Figure S4**), and B-cell count (*p* = 0.0026, **Figure S4**) were significantly higher in ESCC patients with non-PD responses than in those with PD. In contrast, compared to patients with PD, the ESR (*p* = 0.0137, **Figure S4**), hsCRP (*p* = 0.0261, **Figure S4**), LDH (*p* = 0.0303, **Figure S4**), CAR (*p* = 0.0171, **Figure S4**), COP (*p* = 0.0195, **Figure S4**), CLR (*p* = 0.0148, **Figure S4**), and CBR (*p* = 0.0146, **Figure S4**) levels were significantly lower in non-PD patients.

In the GAC cohort, the WBC (*p* = 0.0310, **Figure 2E**), PLT (*p* = 0.0413, **Figure 2F**), neutrophil (*p* = 0.0270, **Figure 2G**), monocyte (*p* = 0.0215, **Figure 2H**), and CD8^+^CD28^+^T (*p* = 0.0121, **Figure 2I**) cell counts significantly increased in patients with PR. Eosinophil (*p* = 0.0284, **Figure S4**) and ELR (*p* = 0.0109, **Figure S4)** levels were significantly higher in non-PD patients than in PD patients.

### 3.4 Analysis of the circulating biomarkers as predictors of chemotherapeutic response

We performed ROC analysis of circulating parameters to identify patients with different responses to chemotherapy (PR/non-PR and non-PD/PD) in the ESCC and GAC cohorts.

**Figure 3:**
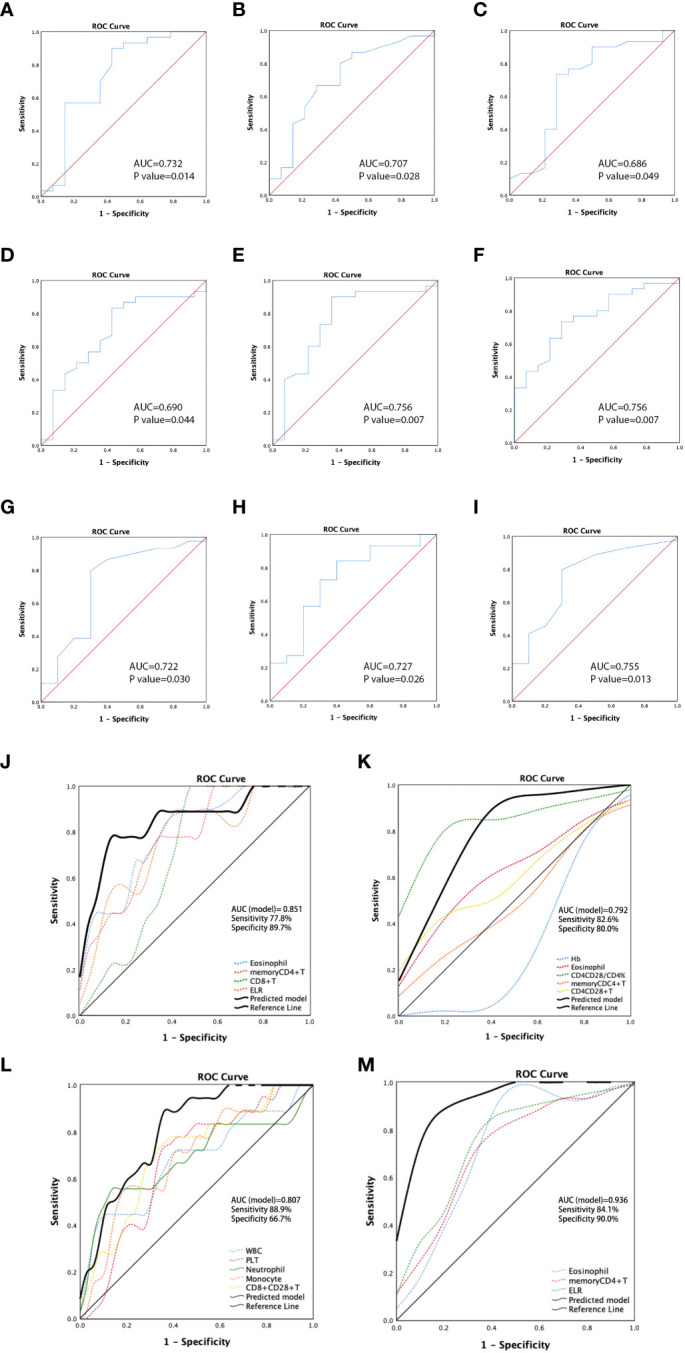
Receiver operating characteristic (ROC) curves for predicting non-PD in patients with ESCC by hemoglobin (Hb) **(A)**, eosinophil **(B)**, CD4^+^CD28^+^T/CD4^+^T percentage **(C)**, CD8^+^CD38^+^T/CD8^+^T percentage **(D)**, memory CD4^+^T **(E)**, and CD4^+^CD28^+^T **(F)**. ROC curves demonstrating the ability to discriminate patients with non-PD and those with PD in patients with GAC according to values of eosinophil **(G)**, memory CD4^+^T **(H)**, and ELR **(I)**. ROC curves for predictive models showing the predicting potential of PR vs. non-PR **(J)** and non-PD vs. PD **(K)** for patients with ESCC, and the predicting ability of PR vs. non-PR **(L)** and non-PD vs. PD **(M)** for patients with GAC. ESCC, esophageal squamous carcinoma; GAC, gastric adenocarcinoma.

In the ROC analysis, the areas under the curve (AUCs) for discriminating the ESCC cohort patients with PR from those with non-PR according to the eosinophil count, CD8^+^T, memory CD4^+^T cell count, and ELR were 0.797 (*p* = 0.007), 0.775 (*p* = 0.012), 0.749 (*p*=0.022), and 0.757 (*p* = 0.018), respectively **(** **Figure S5 A-D**). The ROC curve analysis revealed a significantly better chemotherapeutic response in patients with ESCC with higher levels of these predictors. The optimal cutoff value for eosinophils was calculated as 0.125, with a sensitivity of 77.8% and a specificity of 68.6%. The cutoff values for CD8^+^T and memory CD4^+^T cells were 427 and 381, respectively. The sensitivity for CD8^+^T was 77.8%, and the specificity was 74.3%. The sensitivity for memory CD4^+^T cells was 100%, and the specificity was 54.3%. The cutoff value for ELR was 0.075, with a sensitivity of 88.9% and a specificity of 57.1%. Hemoglobin (Hb, **Figure 3A**), eosinophils (**Figure 3B**), CD4^+^CD28^+^T/CD4^+^T percentage (**Figure 3C**), CD8^+^CD38^+^T/CD8^+^T percentage (**Figure 3D**), memory CD4^+^T (**Figure 3E**), and CD4^+^CD28^+^T (**Figure 3F**) were all identified as predictors for distinguishing patients with non-PD from those with PD in the ESCC cohort. There was better disease control in patients with higher levels of these predictors, except for CD8^+^CD38^+^T/CD8^+^T cells. The cutoff value of Hb was calculated as 114.5 g/L with an AUC of 0.732 (*p* = 0.014), a sensitivity of 90%, and a specificity of 57.1%. The cutoff for eosinophil count was 0.115 with an AUC of 0.707 (*p* = 0.028), a sensitivity of 66.7%, and a specificity of 71.4%. The cutoff for CD4^+^CD28^+^T/CD4^+^T cell percentage was 92.65%, with an AUC of 0.686 (*p* = 0.049), a sensitivity of 73.3%, and a specificity of 71.4%. The cutoff for CD8^+^CD38^+^T/CD8^+^T cell percentage was 43.2%, with an AUC of 0.690 (*p* = 0.044), a sensitivity of 83.3%, and a specificity of 57.1%. The cutoff for memory CD4^+^T cells was 282, with an AUC of 0.756 (*p* = 0.007), a sensitivity of 90%, and a specificity of 64.3%. The cutoff value for CD4^+^CD28^+^T cells was 486, with an AUC of 0.756 (*p* = 0.007), a sensitivity of 73.3%, and a specificity of 71.4%.

In the GAC cohort, higher levels of WBC, PLT, neutrophils, monocytes, and CD8^+^CD28^+^T cells were found to be predictors of PR to chemotherapy. In ROC analysis, the AUCs for discriminating PR from non-PR according to these markers were 0.680 (*p* = 0.033), 0.671 (*p* = 0.042), 0.685 (*p* = 0.028), 0.692 (*p* = 0.022), and 0.709 (*p* = 0.013), respectively **(** **Figures S5E–I**). The cutoff values were 8.415 (sensitivity: 44.4%; specificity: 91.7%), 255 (sensitivity: 72.2%; specificity: 66.7%), 5.895 (sensitivity: 55.6%; specificity: 88.9%), 0.445 (sensitivity: 55.6%; specificity: 80.6%), and 136.5 (sensitivity: 77.8%, specificity: 63.9%), respectively. Additionally, high levels of eosinophils **(** **Figure 3G**), memory CD4^+^T cells **(** **Figure 3H**), and ELR **(** **Figure 3I**) were predictors of disease control (non-PD). The cutoff values were 0.055 (*p* = 0.030, sensitivity: 79.5%, specificity: 70%), 251 (*p* = 0.026, sensitivity: 84.1%, specificity: 60%), and 0.045 (*p* = 0.013, sensitivity: 79.5%, specificity: 70%), respectively.

Based on the ROC analysis of the predictors of PR/non-PR and non-PD/PD in cohorts with ESCC or GAC, we established multi-analyte predictive models with a combination of significant circulating markers to identify better indicators for predicting chemotherapeutic response in the ESCC and GAC cohorts, respectively. The sensitivity and specificity of each combined model demonstrated better predictive ability than a single parameter. The models showed that combined detection exhibited a good ability to distinguish PR from non-PR in patients with ESCC (AUC = 0.851, *p* = 0.002, **Figure 3J**), as well as to distinguish non-PD from PD (AUC = 0.792, *p* < 0.001, **Figure 3K**). For GAC, ROC analysis of the predictive models showed the predictive potential of PR vs. non-PR (AUC = 0.807, *p* < 0.001, **Figure 3L**) and non-PD vs. PD (AUC = 0.936, *p* < 0.001, **Figure 3M**).

### 3.5 Analysis of the prognostic impact of the circulating biomarkers on PFS and OS

We further evaluated the prognostic value of the circulating parameters in patients with ESCC and GAC. The corresponding optimal cutoff values for the parameters were determined by ROC curves based on the limits of normal values or median values. The participants were divided into subgroups according to the level of parameters or clinicopathological features. We performed univariate and multivariate analyses of the survival outcomes in patients with ESCC and GAC.

**Figure 4:**
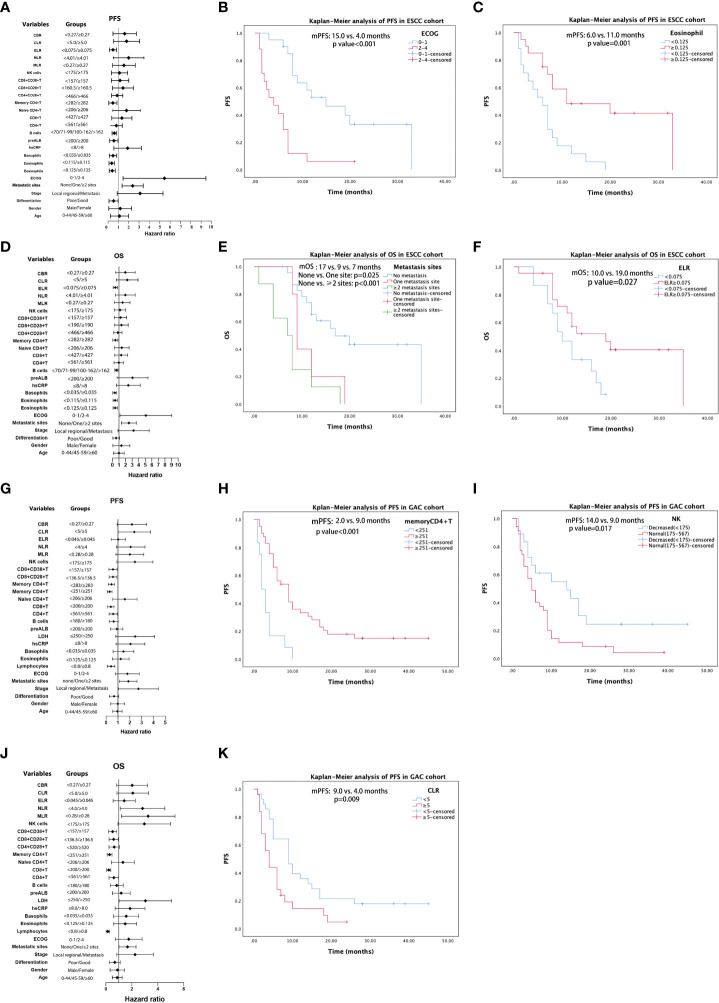
Results of univariable analysis for progression-free survival (PFS) and overall survival (OS) for patients with ESCC **(A, D)** and AGC **(G, J)**, respectively. PFS curves by Kaplan–Meier analyses for patients with ESCC by ECOG **(B)** and eosinophil **(C)**. OS curves of patients with ESCC by metastatic sites **(E)** and ELR **(F)**. PFS Kaplan–Meier curves of patients with GAC by memory CD4^+^T **(H)**, NK cells **(I)**, and CLR **(K)**.

For patients in the ESCC cohort, the univariate analysis showed that tumor differentiation (poor vs. good), stage (local regional vs. distant metastasis), metastatic sites (none vs. one vs. ≥two sites), ECOG (0–1 vs. 2–4), eosinophil count (<0.115 vs. ≥0.115; <0.125 vs. ≥0.125), basophil count (<0.035 vs. ≥0.035; determined as median value), prealbumin level (normal vs. decreased<200), B-cell count (compared by quartile: <70 vs. 71–99 vs. 100–162 vs. >162), memory CD4^+^T cell count (<282 vs. ≥282), and ELR (<0.075 vs. ≥0.075) were associated with PFS (**Table 2**, **Figure 4A**). Eleven factors (*p* < 0.15) were included in the multivariate analyses. Multivariate analysis of ESCC patient outcomes further showed that worse ECOG status was independently associated with worse PFS (HR: 4.818, 95% CI: 2.076–11.184, *p* < 0.001). Higher eosinophil levels were independently and significantly correlated with better PFS (HR: 0.276, 95% CI: 0.120–0.636, *p* = 0.003) (**Table 2**). The PFS curves for patients with ESCC with different ECOG statuses and eosinophil levels were calculated using the Kaplan–Meier method (**Figures 4B, C**). According to univariate Cox analysis, differentiation, stage, metastatic sites, ECOG, eosinophil counts, basophil counts, hsCRP level (normal vs. increased >8), prealbumin level, B-cell count, memory CD4^+^T cell count, and ELR were associated with OS (**Table 3**, **Figure 4D**). However, only the number of metastatic sites (HR: 2.092, 95% CI: 1.307–3.351, *p* = 0.002) and ELR (HR: 0.379, 95% CI: 0.161–0.893, *p* = 0.027) were confirmed as independent predictors of OS in the multivariate analysis (**Table 3**). Thus, the more the number of metastatic sites, the worse the OS. Higher ELR was significantly correlated with better OS. The Kaplan–Meier curves for the OS of patients with ESCC according to the prognostic factors are shown in **Figures 4E, F**.

**Table 2:** Univariate and multivariate analysis of PFS in ESCC cohort.

Variables	Univariate			Multivariate analysis		
	HR	95%CI	P	HR	95%CI	P
**Age**						
0-44 vs. 45-59 vs. ≥60	0.877	0.369-2.081	0.765			
**Gender**						
Male vs Female	0.838	0.290-2.419	0.744			
**Differentiation**						
Poor vs. good	0.418	0.169-1.035	0.059	NA	NA	0.088
**Stage**						
Local regional vs. metastatic	2.577	1.200-5.533	0.015	NA	NA	0.175
**Metastatic sites**						
None vs. One vs. ≥two sites	2.235	1.438-3.475	<0.001	NA	NA	0.248
**ECOG**						
0-1 vs. 2-4	4.48	2.034-9.868	<0.001	4.818	2.076-11.184	<0.001
**Esophils**						
<0.115 vs. ≥0.115	0.34	0.157-0.736	0.006	NA	NA	0.315
<0.125 vs. ≥0.125	0.289	0.130-0.642	0.002	0.276	0.120-0.636	0.003
**Basophils**						
<0.035 vs. ≥0.035	0.396	0.178-0.878	0.023	NA	NA	0.473
**hsCRP**						
≤8 vs. >8	1.587	0.750-3.358	0.227			
**preALB**						
<200 vs. ≥200	0.471	0.221-1.005	0.052	NA	NA	0.976
**B cells**						
<70 vs. 71-99 vs.	0.587	0.401-0.857	0.006	NA	NA	0.436
100-162 vs.>162						
**CD4+T**						
<561 vs. ≥561	0.677	0.321-1.431	0.307			
**CD8+T**						
<427 vs. ≥427	1.105	0.507-2.408	0.802			
**Naive CD4+T**						
<206 vs. ≥206	1.461	0.656-3.251	0.353			
**Memor y CD4+T**						
<282 vs. ≥282	0.364	0.145-0.913	0.031	NA	NA	0.874
**CD4+CD28+T**						
<466 vs. ≥466	1.025	0.283-1.341	0.222			
**CD8+CD28+T**						
<160.5 vs. ≥160.5	1.207	0.568-2.565	0.625			
**CD8+CD38+T**						
<157 vs. ≥157	0.96	0.442-2.085	0.919			
**NK cells**						
<175 vs. ≥175	0.929	0.439-1.970	0.849			
**MLR**						
<0.27 vs. ≥0.27	1.281	0.599-2.738	0.524			
**NLR**						
<4.01 vs. ≥4.01	1.63	0.734-3.623	0.230			
**ELR**						
<0.075 vs. ≥0.075	0.398	0.183-0.866	0.020	NA	NA	0.707
**CLR**						
<5.0 vs. ≥5.0	1.502	0.713-3.165	0.285			
**CBR**						
<0.27 vs. ≥0.27	1.346	0.634-2.857	0.439			

NA, not applicable.

**Table 3:** Univariate and multivariate analysis of OS in ESCC cohort.

Variables	Univariate analysis			Multivariate analysis		
	HR	95%CI	P	HR	95%CI	P
**Age**					
0-44 vs. 45-59 vs. ≥60	0.781	0.325-1.878	0.581			
**Gender**					
Male vs. Female	0.965	0.330-2.818	0.948			
**Differ entiation**					
Poor vs. good	0.428	0.164-1.120	0.084	NA	NA	0.125
**Stage**					
Locoregional vs. metastatic	2.641	1.197-5.830	0.016	NA	NA	0.848
**Metastatic sites**					
None vs. One vs. ≥two sites	2.344	1.483-3.704	<0.001	2.092	1.307-3.351	0.002
**ECOG**					
0-1 vs. 2-4	4.082	1.765-9.440	0.001	3.624	1.511-8.695	0.004
**Esophils**					
<0.115 vs. ≥0.115	0.353	0.158-0.786	0.011	NA	NA	0.772
<0.125 vs. ≥0.125	0.344	0.152-0.775	0.01	NA	NA	0.567
**Basophils**					
<0.035 vs. ≥0.035	0.29	0.119-0.706	0.006	NA	NA	0.232
**hsCRP**					
≤8 vs. >8	1.974	0.884-4.409	0.097	NA	NA	0.597
**preALB**					
<200 vs. ≥200	2.474	1.081-5.665	0.032	NA	NA	0.194
**B cells**					
<70 vs. 71-99 vs.	0.586	0.400-0.859	0.006	NA	NA	0.214
100-162 vs.>162						
**CD4+T**					
<561 vs. ≥561	0.796	0.359-1.762	0.573			
**CD8+T**					
<427 vs. ≥427	1.105	0.507-2.408	0.802			
**Naive CD4+T**					
<206 vs. ≥206	1.145	0.493-2.658	0.753			
**Memor y CD4+T**					
<282 vs. ≥282	0.348	0.138-0.876	0.025	NA	NA	0.411
**CD4+CD28+T**					
<466 vs. ≥466	0.676	0.298-1.533	0.348			
**CD8+CD28+T**					
<190 vs. ≥190	1.13	0.509-2.508	0.764			
**CD8+CD38+T**					
<157 vs. ≥157	1.021	0.458-2.275	0.960			
**NK cells**					
<175 vs. ≥175	0.959	0.435-2.116	0.918			
**MLR**					
<0.27 vs. ≥0.27	1.255	0.563-2.800	0.578			
**NLR**					
<4.01 vs. ≥4.01	1.588	0.699-3.604	0.269			
**ELR**					
<0.075 vs. ≥0.075	0.421	0.186-0.953	0.038	0.379	0.161-0.893	0.027
**CLR**					
<5.0 vs. ≥5.0	1.844	0.835-4.072	0.13	NA	NA	0.860
**CBR**					
<0.27 vs. ≥0.27	1.597	0.705-3.619	0.262			

NA, not applicable.

In univariate analysis, differentiation, stage, metastatic sites, ECOG score, lymphocyte count (decreased < 0.8 vs. normal), hsCRP level, LDH level (normal vs. increased > 250), B-cell count (decreased < 180 vs. normal), CD4^+^T count (decreased < 561 vs. normal), CD8^+^T count (decreased < 200 vs. normal), memory CD4^+^T count (decreased < 251 vs. normal), CD8^+^CD28^+^T count (<136.5 vs. ≥136.5), CD8^+^CD38^+^T count (<157 vs. ≥157), and NK cell count (decreased < 175 vs. ≥175) were significant potential predictive factors for PFS in GAC patients. When we confirmed the cutoff values based on the near medians, MLR (<0.28 vs. ≥0.28), NLR (<4.0 vs. ≥4.0), CLR (<5.0 vs. ≥5.0), and CBR (<0.27 vs. ≥0.27) demonstrated potential predictive significance for PFS in univariate analysis (**Table 4**, **Figure 4G**). Multivariate analysis performed on the GAC cohort showed that differentiation (HR: 0.041, 95% CI: 0.200–0.803, *p* = 0.010), memory CD4^+^T count (HR: 0.304, 95% CI: 0.137–0.675, *p* = 0.003), NK cell count (HR: 2.302, 95% CI: 1.044–3.953, *p* = 0.037), and CLR (HR: 2.070, 95% CI: 1.024–4.186, *p* = 0.043) were significant independent prognostic factors for PFS (**Table 4**). We display the Kaplan–Meier curves of patients with GAC between subgroups with different levels of memory CD4^+^T cell count (**Figure 4H**), NK cell count (**Figure 4I**), and CLR (**Figure 4K**) in **Figure 4**. Based on the univariate analysis, 18 parameters were identified as prognostic factors associated with OS (**Table 5**, **Figure 4J**). Multivariate analysis with the selected markers using the Cox regression model showed that total lymphocyte counts (HR: 0.260, 95% CI: 0.086–0.783, *p* = 0.017), CD8^+^T count (HR: 0.405, 95% CI: 0.165–0.997, *p* = 0.049), NK cell count (HR: 3.395, 95% CI: 1.592–7.238, *p* = 0.002), and MLR (HR: 3.076, 95% CI: 1.488–6.360, *p* = 0.002) were identified as independent factors associated with OS (**Table 5**). GAC patients with higher levels of lymphocytes and CD8^+^T cells had better OS than those with lower levels. Nevertheless, higher NK and MLR may predict worse outcomes. The Kaplan–Meier curves for the OS of patients with GAC according to these prognostic factors are shown in **Figure S6**.

**Table 4:** Univariate and multivariate analysis of PFS in GAC cohort.

Variables	Univariate analysis			Multivariate analysis		
	HR	95%CI	P	HR	95%CI	P
**Age**					
0-44 vs. 45-59 vs. ≥60	0.908	0.600-1.376	0.651			
**Gender**					
Male vs. Female	0.875	0.472-1.624	0.672			
**Differ entiation**					
Poor vs. good	0.594	0.318-1.112	0.103	0.401	0.200-0.803	0.010
**Stage**					
Locoregional vs. metastatic	2.381	1.239-4.575	0.009	NA	NA	0.539
**Metastatic sites**					
None vs. One vs. ≥two sites	1.791	1.206-2.659	0.004	NA	NA	0.263
**ECOG**					
0-1 vs. 2-4	1.603	0.890-2.890	0.116	NA	NA	0.741
**Lymphocytes**					
<0.8 vs. ≥0.8	0.337	0.146-0.776	0.011	NA	NA	0.516
**Esophils**					
<0.125 vs. ≥0.125	1.109	0.614-2.006	0.731			
**Basophils**					
<0.035 vs. ≥0.035	1.294	0.691-2.421	0.421			
**hsCRP**					
≤8 vs. >8	1.825	0.985-3.383	0.056	NA	NA	0.499
**LDH**					
≤250 vs. >250	2.1	1.038-4.248	0.039	NA	NA	0.953
**preALB**					
<200 vs. ≥200	0.812	0.450-1.464	0.488			
**B cells**					
<180 vs. ≥180	0.57	0.310-1.048	0.071	NA	NA	0.684
**CD4+T**					
<561 vs. ≥561	0.532	0.292-0.970	0.039	NA	NA	0.384
**CD8+T**					
<200 vs. ≥200	0.34	0.166-0.697	0.003	NA	NA	0.832
**Naive CD4+T**					
<206 vs. ≥206	1.371	0.691-2.718	0.366			
**Memory CD4+T**					
<251 vs. ≥251	0.257	0.126-0.524	<0.001	0.304	0.137-0.675	0.003
<283 vs. ≥283	0.405	0.220-0.744	0.004	NA	NA	0.58
**CD8+CD28+T**					
<136.5 vs. ≥136.5	0.522	0.289-0.944	0.032	NA	NA	0.993
**CD8+CD38+T**					
<157 vs. ≥157	0.52	0.281-0.960	0.037	NA	NA	0.634
**NK cells**					
<175 vs. ≥175	2.122	1.100-4.091	0.025	2.032	1.044-3.953	0.037
**MLR**					
<0.28 vs. ≥0.28	1.783	0.969-3.283	0.063	NA	NA	0.475
**NLR**					
<4.0 vs. ≥4.0	1.844	1.002-3.393	0.049	NA	NA	0.434
**ELR**					
<0.045 vs. ≥0.045	0.91	0.495-1.674	0.762			
**CLR**					
<5.0 vs. ≥5.0	2.121	1.166-3.859	0.014	2.07	1.024-4.186	0.043
**CBR**					
<0.27 vs. ≥0.27	1.946	1.071-3.534	0.029	NA	NA	0.702

NA, not applicable.

**Table 5:** Univariate and multivariate analysis of OS in GAC cohort.

Variables	Univariate analysis			Multivariate analysis		
	HR	95%CI	P	HR	95%CI	P
**Age**					
0-44 vs. 45-59 vs. ≥60	0.816	0.522-1.276	0.373			
**Gender**					
Male vs. Female	0.767	0.391-1.503	0.439			
**Differentiation**					
Poor vs. good	0.596	0.307-1.155	0.125	NA	NA	0.058
**Stage**					
Locoregional vs. metastatic	1.955	1.011-3.780	0.046	NA	NA	0.208
**Metastatic sites**					
None vs. One vs. ≥two sites	1.583	1.061-2.362	0.025	NA	NA	0.148
**ECOG**					
0-1 vs. 2-4	1.557	0.843-2.877	0.157			
**Lymphocytes**					
<0.8 vs. ≥0.8	0.115	0.045-0.298	<0.001	0.26	0.086-0.783	0.017
**Esophils**					
<0.125 vs. ≥0.125	1.318	0.710-2.447	0.381			
**Basophils**					
<0.035 vs. ≥0.035	1.366	0.714-2.616	0.346			
**hsCRP**					
≤8 vs. >8	1.632	0.854-3.116	0.138	NA	NA	0.432
**LDH**					
≤250 vs. >250	2.598	1.282-5.263	0.008	NA	NA	0.994
**preALB**					
<200 vs. ≥200	1.048	0.570-1.927	0.88			
**B cells**					
<180 vs. ≥180	0.737	0.395-1.377	0.339			
**CD4+T**					
<561 vs. ≥561	0.54	0.286-1.017	0.057	NA	NA	0.381
**CD8+T**					
<200 vs. ≥200	0.187	0.087-0.402	<0.001	0.405	0.165-0.997	0.049
**Naive CD4+T**					
<206 vs. ≥206	1.115	0.547-2.275	0.764			
**Memor y CD4+T**					
<251 vs. ≥251	0.246	0.123-0.492	<0.001	NA	NA	0.772
**CD4/CD8**					
≤2.13 vs. >2.13	2.146	1.101-4.184	0.025	NA	NA	0.116
**CD4+CD28+T**					
<520 vs. ≥520	0.56	0.290-1.081	0.084	NA	NA	0.532
**CD8+CD28+T**					
<136.5 vs. ≥136.5	0.531	0.287-0.982	0.044	NA	NA	0.783
**CD8+CD38+T**					
<157 vs. ≥157	0.458	0.240-0.873	0.018	NA	NA	0.608
**NK cells**					
<175 vs. ≥175	2.5	1.204-5.191	0.014	3.395	1.592-7.238	0.002
**MLR**					
<0.28 vs. ≥0.28	2.827	1.449-5.512	0.002	3.076	1.488-6.360	0.002
**NLR**					
<4.0 vs. ≥4.0	2.473	1.303-4.694	0.006	NA	NA	0.169
**ELR**					
<0.045 vs. ≥0.045	1.239	0.654-2.345	0.511			
**CLR**					
<5.0 vs. ≥5.0	1.834	0.990-3.399	0.054	NA	NA	0.573
**CBR**					
<0.27 vs. ≥0.27	1.793	0.969-3.317	0.063	NA	NA	0.318

NA, not applicable.

## 4 Discussion

In the current study, we sought to explore whether inflammatory- and immune-related indicators clinically influence the treatment efficacy or survival in patients receiving chemotherapy for UGI cancer. To the best of our knowledge, few studies have simultaneously assessed these circulating parameters. Circulating inflammatory and lymphocyte subsets were associated not only with clinicopathological features but also with chemotherapeutic response and prognosis. We constructed four predictive models using a combination of different parameter types that yielded satisfactory prediction abilities.

Previous studies have shown that PLTs are recruited into the tumor environment from the peripheral blood circulation and are involved in inflammatory reactions. In addition, they are activated by inducing factors, such as adenosine diphosphate (ADP), which is released by cancer cells (18, 19). Our finding that significantly higher PLT counts were related to worse ECOG scores supports this theory because poor performance status is commonly clinically observed in the late stages of cancer. Lymphocytes are crucial immune cells. They are believed to possess anticancer properties by inhibiting cell growth and migration in tumors, including GC. This process demonstrates the critical role of lymphocytes in host and antitumor immune responses (20). Evidence indicates that circulating lymphocyte counts are correlated with complete response to neoadjuvant chemoradiotherapy in patients with rectal cancer (21). Our results showing that decreased lymphocytes were associated with worse UGI cancer status similarly demonstrate the impact of lymphocytes on immunity and cancer progression. Thus, we speculate that combined parameters based on lymphocytes may be crucial for achieving a better response to cancer therapy. The absolute neutrophil count has been positively associated with advanced cancer stage in some studies (22, 23), consistent with our results. Neutrophils are commonly represented as a reflection of tumor burden because of their contribution to tumor-related inflammation. Several studies have indicated that the crosstalk between inflammatory mediators in cancer cells and the microenvironment promotes metastasis (24), suggesting that cancer-related inflammation promotes cancer progression. Serum C-reactive protein (CRP) is an acute-phase reactant that mediates a systemic inflammatory response. Various reports have regarded CRP levels as a strong indicator of poor prognosis in patients with cancer, including EC (25). ESR and CRP were both observed to be associated with advanced stages and worse ECOG status in our study, verifying the ability of these inflammatory parameters as biomarkers to indicate cancer severity; this may, thus, help clinicians make clinical decisions. Increased baseline LDH level was also associated with advanced cancer stage and worse performance status and has been extensively studied in multiple tumors (26–28). Albumin and prealbumin levels are important indicators of nutritional status (29). Poor nutritional status is commonly observed in patients with tumors, especially UGI tumors, which directly affect the nutritional intake of patients. This may be a reasonable explanation for our results, indicating that these two parameters are related to more advanced tumors and worse physical performance.

Immune conditions are closely associated with cancer development. Therefore, peripheral blood immune parameters may serve as valuable indicators. In our study, higher levels of CD8^+^CD28^+^T cells were observed in the 45–59-year group compared with those in the other age groups. Recent studies have indicated that lymphocyte subsets are related to the age of patients with cancer. According to a previous report, the loss of CD28 expression on T cells was significantly associated with the aging of the immune system (30); this report identified a decreasing trend of CD8^+^CD28^+^T cells with age. There were only eight patients aged 0–44 years; thus, the presumed high level of CD8^+^CD28^+^T cells in this group was not significant, which partly explained the highest level in the 45–59-year group. We observed higher levels of memory CD4^+^, CD4^+^CD28^+^T, and CD8^+^CD28^+^T cells in patients with ESCC than in those with GAC. Few studies have evaluated the differences in lymphocyte subsets among different cancer types. In our study, the proportion of patients aged >59 years with GAC was significantly higher than that of patients with ESCC. Therefore, we deduced that elderly patients may have a worse immune status. Advanced stage and metastatic sites were negatively associated with B, CD4^+^T, memory CD4^+^T, and CD4^+^CD28^+^T cell levels. This suggests that immune function is partially damaged by the advancing status of cancer, which causes the rapid proliferation of cancer cells. Although markedly more attention has been focused on T cells in the field of immuno-oncology in recent years, recent studies have also associated human cancer B cells with antitumor immunity. B cells have the capacity to enhance T-cell responses and cross-present antigens to T cells, which could be especially important in tertiary lymphoid structures (TLS). TLS are composed of various immune cells with lymph node-like features that are considered crucial for producing and sustaining immune responses. B cells in the TLS secrete antibodies that recognize tumor-associated antigens (31). In some studies, high expression of B-cell-related markers was associated with a significantly better prognosis (32). However, some regulatory B cell (Breg) subsets have been identified to participate in tumor escape from immunosurveillance. Our study showed that the levels of B cells were significantly lower in patients with distant metastatic tumors or worse metastatic conditions, compared with local advanced tumors and single metastatic site, respectively, partly reflecting the association between B cells and tumors. CD4^+^T cells exhibit remarkable functional diversity and various subsets with specific properties. Cytotoxic cells enhance and sustain the antitumor responses of CD8^+^T cells, while immune-suppressing regulatory T cells inhibit immune responses. Some studies have reported that patients with lung cancer or hepatocellular carcinoma have more CD4^+^T cells than do healthy individuals (33, 34). Memory CD4^+^T cells represent adaptive immune memory (35), which may be induced by memory CD4^+^T cells during a second encounter with pathogens. CD28 is a critical costimulatory molecule that participates as a secondary signal for activating CD4^+^ and CD8^+^T cells to produce an antitumor response. Thus, the elevation of CD4^+^CD28^+^T cells might imply an upregulated antitumor immune response due to the influence of cancer. We observed lower levels of CD4^+^T, memory CD4^+^T, and CD4^+^CD28^+^T cells in distant metastatic UGI cancers than in locally advanced cancers. A trend toward decreased counts of these three markers was also noted in patients with more metastatic sites, suggesting that the severe cancer status might have damaged the immune microenvironment or led to immune escape. NK cells contribute to immunity by secreting cytokines (36). Several studies have revealed that low NK cell counts predict a higher risk of tumors (37).

We demonstrated the relationship between the combined parameters and clinicopathological features. These parameters mostly reflected the inflammatory indicators, peripheral blood cells and immune cells, as expected, and the results were consistent with those in the previous literature (38–40), indicating the feasibility of application of these parameters.

Currently, although immunotherapy has become the preferred treatment for advanced UGI cancer, chemotherapy is still considered the basis for systemic therapy. Because of the unfavorable efficacy and prognosis of ESCC and AGC, it is essential to investigate predictive factors for the efficacy of chemotherapy. It would be an innovative strategy to combine immune parameters along with inflammatory and peripheral blood markers to explore risk models for treatment responses. Tumor-related eosinophilia has been described in some tumors (41–43); however, eosinophilia is still underdiagnosed owing to its low incidence (1–7%) in clinical practice (44, 45). The present study revealed that eosinophil count and ELR were significant predictors of efficacy in ESCC and GAC. Few studies have assessed the relationship between eosinophil count and efficacy; thus, our findings regarding the significance of eosinophils and eosinophil-based parameters are significant. In addition, we discovered that the CD8^+^CD38^+^T/CD8^+^T cell percentage was negatively associated with efficacy in patients with ESCC, contrary to that observed for other predictors. CD38 plays a critical predictive role in CD8^+^T activation and CD4^+^T depletion (46). A high percentage of CD38^+^/CD8^+^T can predict cancer progression and immunosuppressive status, implying that the immune system is activated during cancer development (47). We deduced that the negative prediction of the treatment response association might be related to the mechanisms by which CD38^+^/CD8^+^T cells act on the immune process. Furthermore, considering the immunocytes and inflammatory parameters, along with the blood cells, all of which may be potential biomarkers for treatment response, we applied ROC analysis to construct predictive multi-index models, which verified the prediction efficiency. The predictive ability of these panels indicated that changes in the tumor might be influenced by multiple factors. By utilizing this model, clinicians can develop specific strategies to achieve precise medical treatment for patients with UGI cancer.

In the present study, we showed that lower ECOG scores and higher eosinophil counts are independent prognostic factors for improved PFS in patients with ESCC. ELR and fewer metastatic sites were confirmed as positive prognostic factors for better OS in the ESCC cohort. These results are consistent with those of previous studies verifying that increased tumor-associated eosinophils improve the prognosis of esophageal and colorectal cancers (48). However, circulating lymphocyte subsets have not been identified as prognostic factors for EC, despite some subsets having predictive significance for response to chemotherapy. The functions of lymphocyte subsets are complex and tend to be influenced by multiple factors, including chemotherapeutic drugs and infections, during the course of cancer. This may explain why lymphocyte subsets are not significant predictors of survival. For GC, differentiation, memory CD4+T cells, NK cells, and the CLR were identified as prognostic factors for PFS. Moreover, lymphocyte count, CD8^+^T cells, NK cells, and MLR were identified as independent factors associated with OS, consistent with previous results showing that patients with both increased CD4+ and CD8^+^T cell ratios had superior OS in EC (17). Notably, the number of NK cells was negatively associated with PFS and OS, which is contrary to the association of memory CD4+T and CD8^+^T. NK cells participate in tumor immunosurveillance by monitoring and killing tumor cells in an antigen-independent and antigen-dependent manner (36). Lower NK cell counts are associated with advanced lung cancer (49). The function of NK cells depends on the delicate balance between activating and inhibitory signals from cell receptors. Therefore, although NK cells possess cytotoxic capabilities, the specific activity of each NK cell subset is complex. Although GAC is characterized by heterogeneity, the function of immune cells has prognostic significance. This may provide a rationale for the application of immunotherapy owing to the importance of immune cells in the microenvironment of GC. CLR and MLR were negative prognostic factors for PFS and OS, respectively. A retrospective study showed that CLR is an independent factor for OS in colorectal cancer (50). Current research on CLR has shown that CLR doubles the risk of cancer progression. Several studies have indicated that monocytes are associated with poor prognosis in cancer patients (51). The inhibition of the immune system by promoting tumor angiogenesis and proliferation may be the underlying mechanism (52). In contrast, lymphocytes are associated with a better prognosis in cancers (53). Hence, MLR can be used to quantify survival outcomes in patients with malignancies. Consistent with our results, some results have revealed that patients with GC with low MLR levels had better 5-year OS rates, compared with patients with high MLR levels (23). The cutoff value of 0.27 was close to that of 0.28 in our study. The current study showed that a combination of inflammatory indicators and immune parameters has an effective prognostic value in patients with UGI tumors. Therefore, these markers have the potential for wide use in clinical practice.

This study is a further exploration of the analysis of peripheral blood circulation markers of participants in tumor cohorts in a microbiota-focused prospective study. Our study has several limitations that should be acknowledged. This study was conducted at a single institution, and the sample size was not sufficiently large, restricting the generalization of the results. Individual clinicopathological differences were observed in each group, possibly affecting the efficacy analysis results. Because of the small sample size, we did not analyze the AC from different gastric locations. Further studies are required to expand the sample size of this study. In addition, the variation of blood biomarkers during chemotherapy may be related to treatment response, and we did not collect enough biomarker results after treatment for comparison. Furthermore, the cutoff values of the parameters were determined through ROC analysis; nevertheless, some cutoff values were set according to the median value because these parameters were calculated as not statistically significant by ROC analysis. The inconsistency in the approaches used to determine the cutoff value requires further validation. Finally, the chemotherapeutic regimens were not consistent and need to be further stratified by regimen. Moreover, because the combination of chemotherapy and immunotherapy or targeted therapy has become the preferred strategy, predictive biomarkers of chemotherapeutic response may not be applied alone, which requires further investigation.

## 5 Conclusions

In conclusion, our study revealed that specific lymphocyte subsets and inflammatory parameters can accurately predict the chemotherapeutic response and prognosis. Moreover, eosinophil counts and eosinophil-based parameters demonstrated potential significance in predicting the efficacy and survival of patients with ESCC and AGC. These cell profiles, comprising a potential panel of biomarkers, were combined to stratify patients with UGI cancer into different risk groups, thereby leading to changes in the therapeutic intervention strategy. Because peripheral blood parameters can be obtained through relatively non-invasive and inexpensive testing, these indicators and response-predicting models have promising clinical applications.

## Data availability statement

The original contributions presented in the study are included in the article/**Supplementary Material**. Further inquiries can be directed to the corresponding authors.

## Ethics statement

The studies involving human participants were reviewed and approved by the ethics committee of Peking Union Medical Hospital (JS-2745). The patients/participants provided their written informed consent to participate in this study.

## Author contributions

NL and LG designed the study, analyzed the data, and wrote the main manuscript. YG and LZ contributed to data interpretation and manuscript revision. NL and YG collected the data and conducted the follow-up. CB and YW directed this project. All authors contributed to the article and approved the submitted version.

## Supplementary material

Supplementary Figure 1 Scatter plots demonstrated the comparison of the counts of white blood cells (WBC), eosinophils and basophils between different groups stratified by tumors, differentiation, staging, the number of metastasis sites and ECOG score. ESO, esophageal cancer; GAS, gastric cancer.

Supplementary Figure 2 Scatter plots demonstrated the comparison of the levels of PLT,Neutrophils,ESR,CRP, LDH,ALB,preALB,lymphocyte subsets and combined markers (NLR,PLR,MLR,BLR,PLR,CAR,COP,CBR) between/among groups stratified by different clinicopathological features.

Supplementary Figure 3 Representative images for the flow cytometry gating strategy.

Supplementary Figure 4 Scatter plots demonstrated the comparison of several parameters between groups with different treatment response (non-PD vs. PD) in ESCC cohort **(A–K)** and GAC cohort **(L, M)**, respectively.

Supplementary Figure 5 ROC curve discriminating partial regression (PR) vs. non-partial response (non-PR) in ESCCC (**A–D**) and GAC **(E–I)** groups in separate analysis. **(A)** eosinophil; **(B)** CD8^+^T; **(C)** memory CD4^+^T; **(D)** ELR; **(E)** WBC; **(F)** PLT; **(G)** neutrophil; **(H)** monocyte; **(I)** CD8^+^CD28^+^T.

Supplementary Figure 6 Kaplan–Meier curves for OS of the patients with GAC according to lymphocytes **(A)**, CD8^+^T **(B)**, NK cells **(C)** and MLR **(D)**. GAC, gastric adenocarcinoma.
