# Supplementary figures and images for: Prognostic and predictive significance of circulating biomarkers in patients with advanced upper gastrointestinal cancer undergoing systemic chemotherapy
Source: Front Oncol. 2023 Jun 6;13:1195848. doi: 10.3389/fonc.2023.1195848 (PMC10280739; doi:10.3389/fonc.2023.1195848)

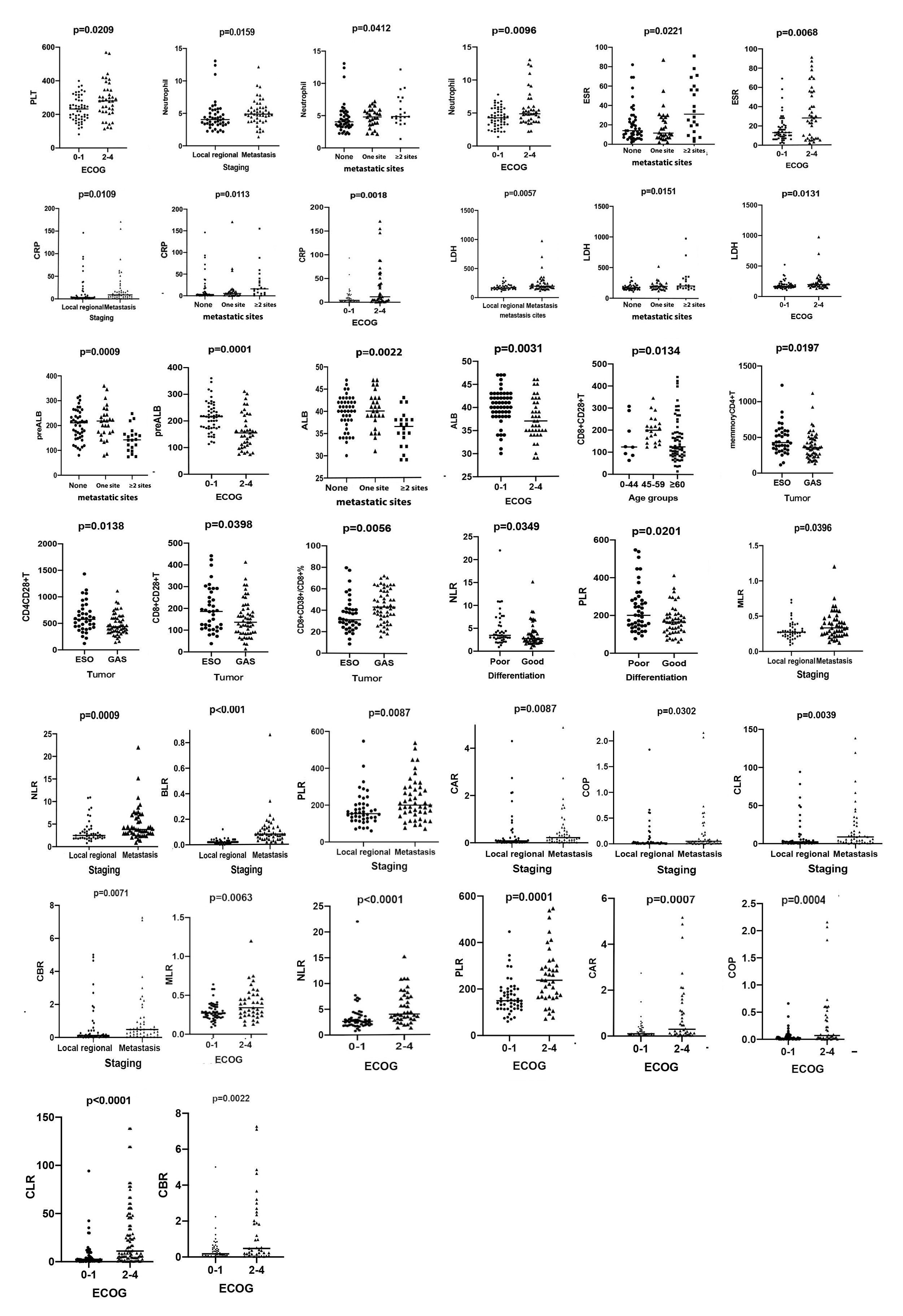

Supplement: Supplementary Figure 2 — Scatter plots demonstrated the comparison of the levels of PLT,Neutrophils,ESR,CRP, LDH,ALB,preALB,lymphocyte subsets and combined markers (NLR,PLR,MLR,BLR,PLR,CAR,COP,CBR) between/among groups stratified by different clinicopathological features. [file Image_2.jpeg]

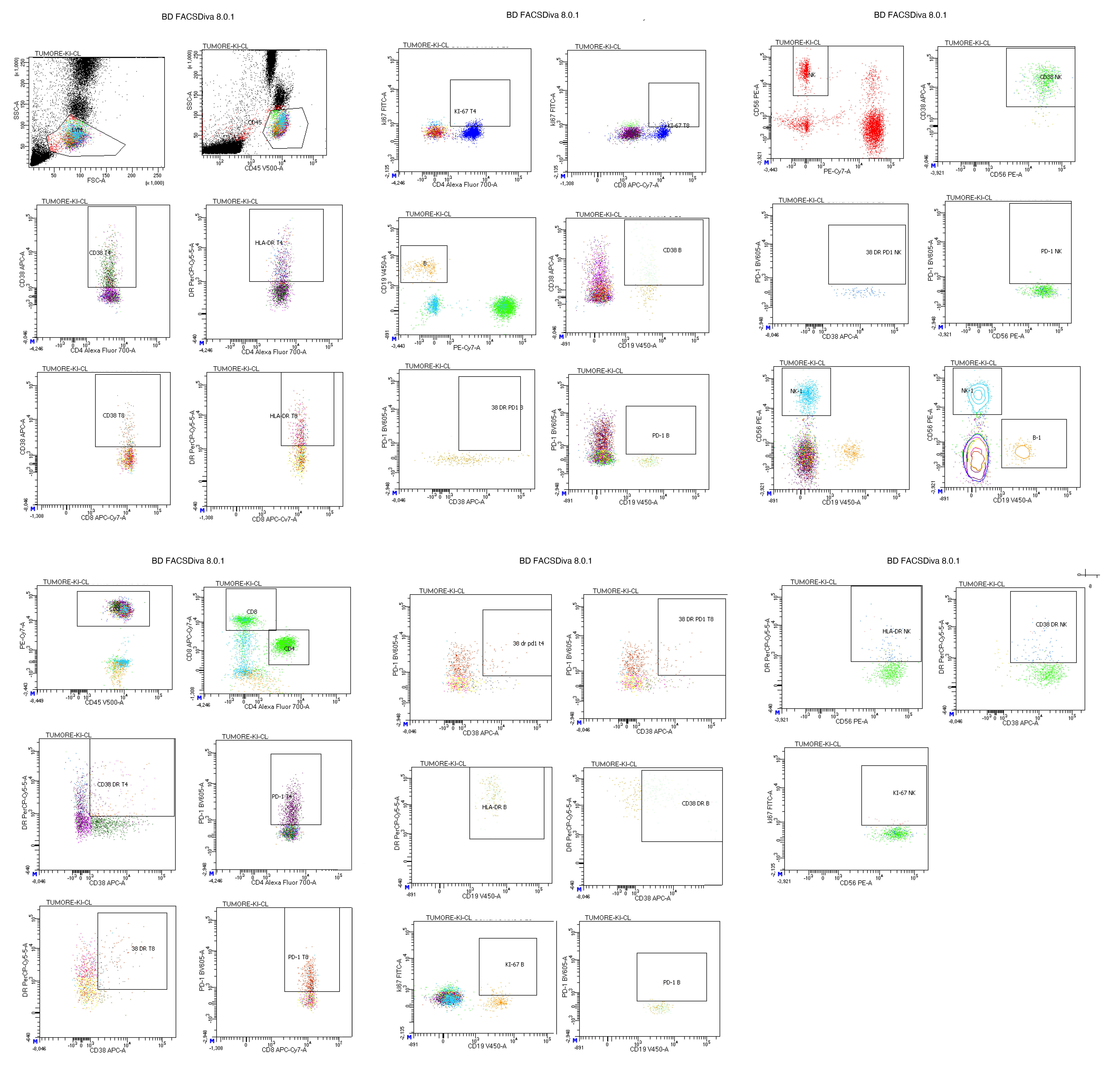

Supplement: Supplementary Figure 3 — Representative images for the flow cytometry gating strategy. [file Image_3.jpeg]

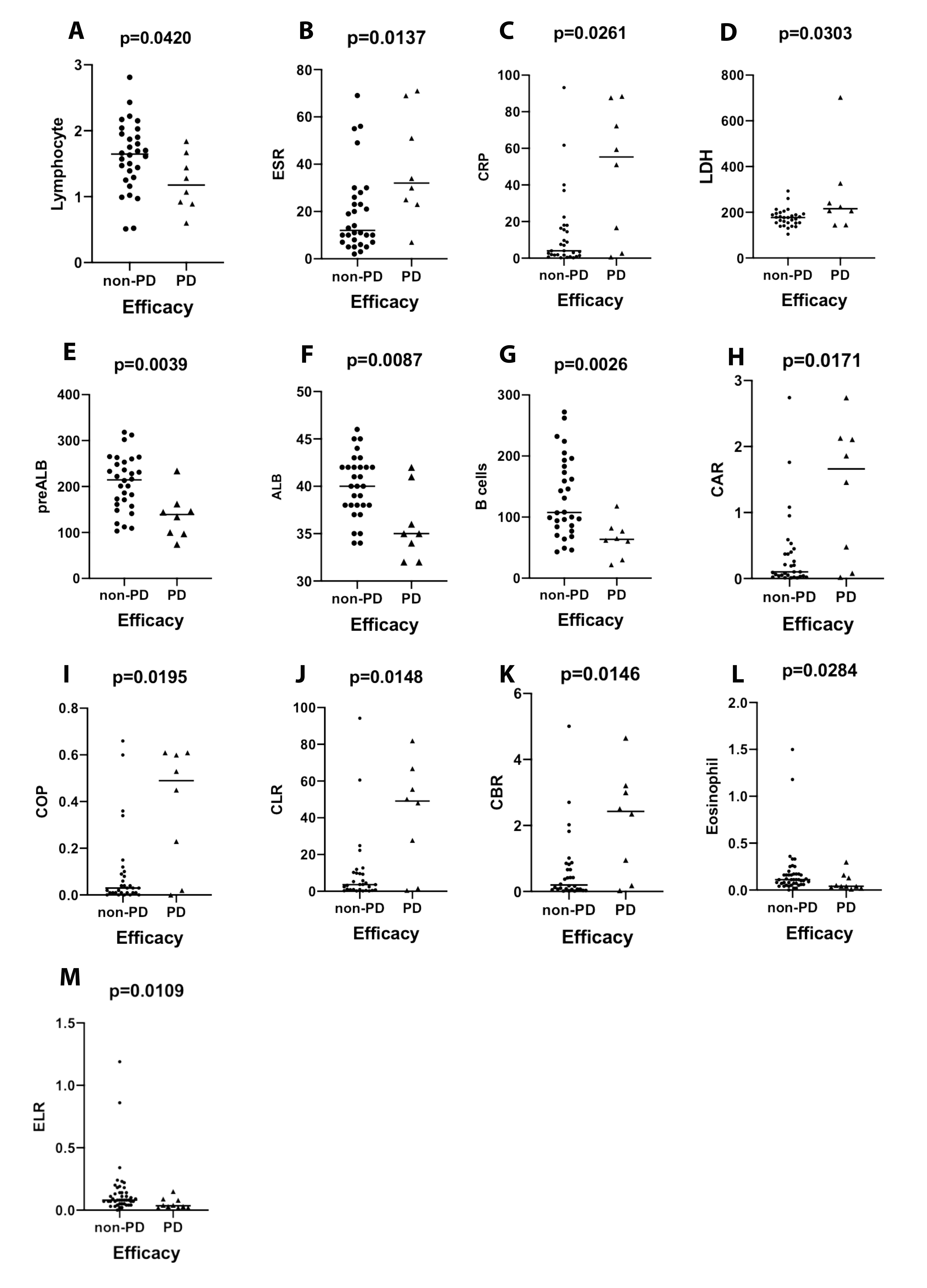

Supplement: Supplementary Figure 4 — Scatter plots demonstrated the comparison of several parameters between groups with different treatment response (non-PD vs. PD) in ESCC cohort (A–K) and GAC cohort (L, M), respectively. [file Image_4.jpeg]

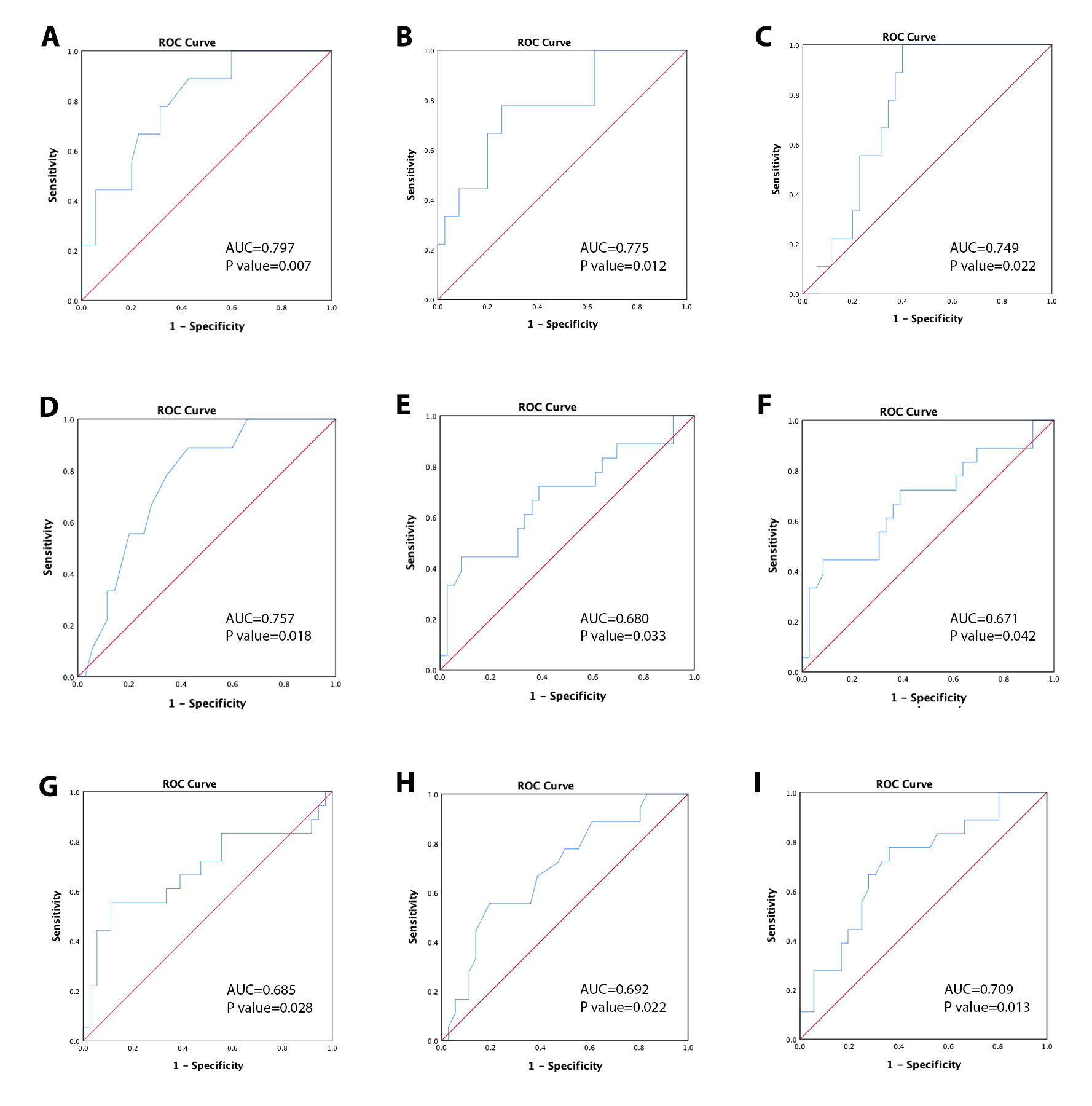

Supplement: Supplementary Figure 5 — ROC curve discriminating partial regression (PR) vs. non-partial response (non-PR) in ESCCC (A–D) and GAC (E–I) groups in separate analysis. (A) eosinophil; (B) CD8+T; (C) memory CD4+T; (D) ELR; (E) WBC; (F) PLT; (G) neutrophil; (H) monocyte; (I) CD8+CD28+T. [file Image_5.jpeg]

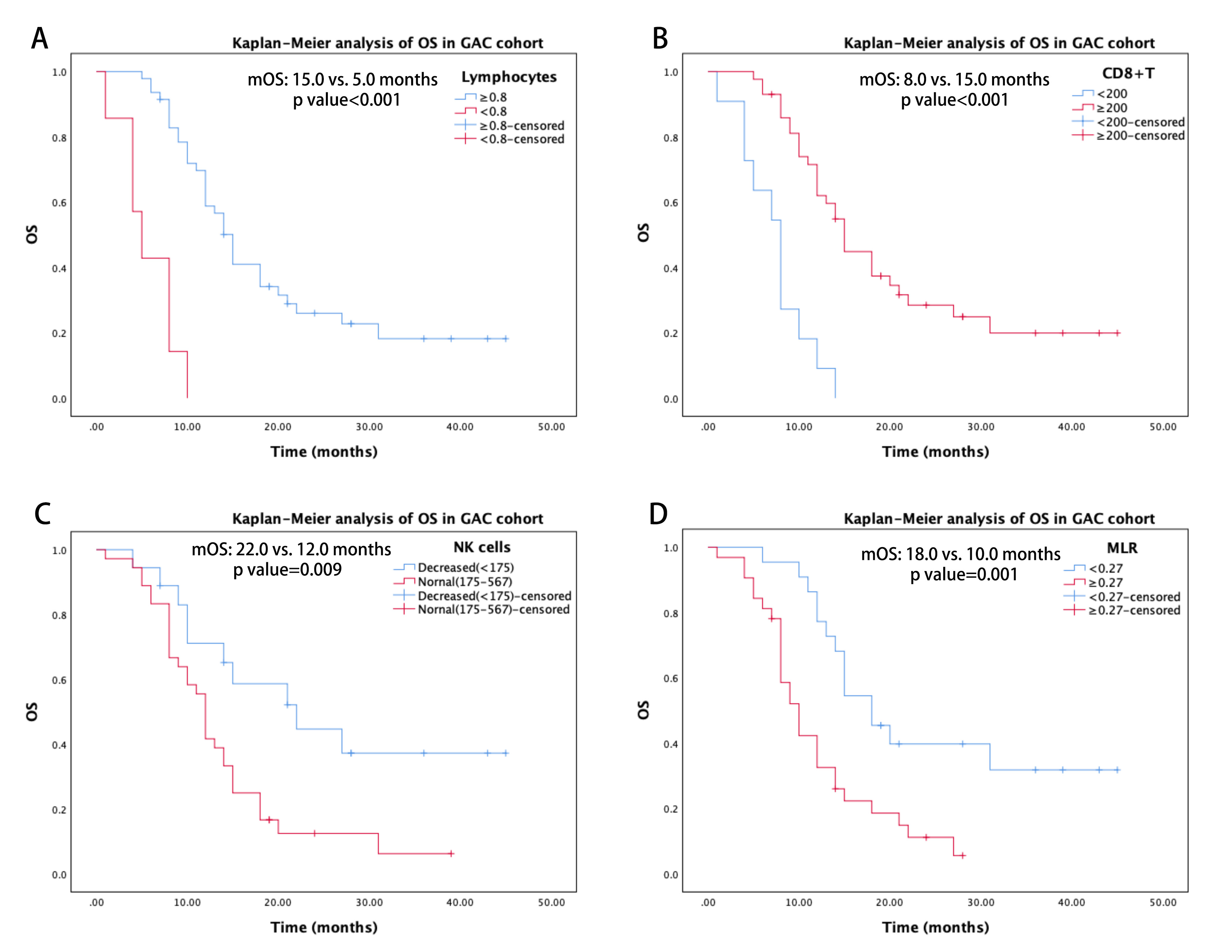

Supplement: Supplementary Figure 6 — Kaplan–Meier curves for OS of the patients with GAC according to lymphocytes (A), CD8+T (B), NK cells (C) and MLR (D). GAC, gastric adenocarcinoma. [file Image_6.jpeg]
