# Supplementary material for: Prognostic and predictive significance of circulating biomarkers in patients with advanced upper gastrointestinal cancer undergoing systemic chemotherapy
Source: Front Oncol. 2023 Jun 6;13:1195848. doi: 10.3389/fonc.2023.1195848 (PMC10280739; doi:10.3389/fonc.2023.1195848)
Supplement: Supplementary file 7 [file Table_1.docx]

**Supplemental Table1. Circulating parameters in UGI cancer patients**

| Circulating parameters | Esophageal cancer (n= 38) | Gastric cancer (n= 54) | Total  （n=92） |
| --- | --- | --- | --- |
| Lymphocytes subsets |  |  |  |
| B cells (cells/ul) | 118.04±65.58 | 110.44±66.91 | 117.90±62.44 |
| NK cells (cells/ul) | 261.29±193.36 | 289.39±213.54 | 194.93±198.59 |
| CD4+T cells (cells/ul) | 636.62±302.96 | 542.15±222.54 | 592.84±260.82 |
| CD8+T cells (cells/ul) | 404.27±199.76 | 373.52±195.06 | 404.67±206.75 |
| Memory CD4+/CD4+(%) | 72.31±14.34 | 73.59±14.65 | 74.80±14.57 |
| Memory CD4+T cells (cells/ul) | 469.51±211.31 | 386.33±187.06 | 435.03±198.25 |
| Naïve CD4+/CD4+(%) | 24.46±11.29 | 24.93±14.35 | 24.54±18.81 |
| Naïve CD4+T cells (cells/ul) | 166.94±118.33 | 137.69±97.71 | 140.67±113.11 |
| CD4+CD28+/CD4+(%) | 93.26±7.76 | 90.51±9.92 | 90.41±10.94 |
| CD4+CD28+T cells (cells/ul) | 612.55±275.83 | 482.30±197.81 | 531.76±231.33 |
| CD8+CD28+/CD8+(%) | 50.65±19.16 | 45.78±16.71 | 49.09±18.45 |
| CD8+CD28+T cells (cells/ul) | 195.74±102.96 | 152.30±84.11 | 182.38±101.95 |
| CD8+CD38+/CD8+(%) | 35.7±16.34 | 44.50±15.23 | 40.17±16.64 |
| CD8+CD38+T cells (cells/ul) | 148.50±122.67 | 162.44±99.97 | 160.67±117.91 |
| CD4+/CD8+ | 1.83±0.94 | 1.76±0.90 | 1.79±1.03 |
| WBC (×10^9^/l) | 7.15±1.86 | 6.79±2.59 | 6.91±2.31 |
| Hb（g/l） | 133.02±17.70 | 113.57±24.11 | 122.27±21.89 |
| PLT (×10^9^/l) | 248.47±93.25 | 258.05±95.38 | 250.20±88.92 |
| Lymphocyte (×10^9^/l) | 1.53±0.52 | 1.34±0.49 | 1.47±0.50 |
| Neutrophil (×10^9^/l) | 4.97±1.92 | 4.85±2.37 | 4.79±2.15 |
| Eosinophil (×10^9^/l) | 0.14±0.10 | 0.16±0.25 | 0.17±0.20 |
| Basophil (×10^9^/l) | 0.04±0.02 | 0.03±0.02 | 0.03±0.02 |
| Monocyte (×10^9^/l) | 0.46±0.38 | 0.41±0.20 | 0.48±0.58 |
| ESR（mm/H） | 23.58±19.91 | 24.36±23.53 | 25.73±21.96 |
| CRP（mg/l） | 20.48±27.97 | 18.08±36.68 | 19.17±29/71 |
| LDH (U/L) | 197.05±95.20 | 218.44±128.65 | 227.82±163.17 |
| ALB (g/l) | 192.61±64.51 | 191.13±66.28 | 193.64±66.73 |
| pALB (mg/l) | 39.13±3.73 | 38.93±4.64 | 39.01±4.41 |

WBC, White Blood Cell; Hb, Hemoglobin; PLT, Platelet; ESR, Erythrocyte Sedimentation Rate; CRP,C-Reactive Protein; LDH, Lactate Dehydrogenase; ALB, Albumin; pALB, Pre-albumin.

**Supplemental Table 2. Circulating combined biomarkers in UGI cancer patients**

| Combined biomarkers | Esophageal cancer (n= 38) | Gastric cancer (n= 54) | Total  （n=92） |
| --- | --- | --- | --- |
| MLR | 0.33±0.16 | 0.33±0.18 | 0.33±1.17 |
| NLR | 3.99±3.42 | 4.33±3.43 | 3.97±2.85 |
| ELR | 0.09±0.05 | 0.13±0.19 | 0.12±0.14 |
| BLR | 0.02±0.01 | 0.02±0.02 | 0.02±0.02 |
| PLR | 183.04±98.28 | 217.09±109.20 | 190.94±96.52 |
| CAR | 0.63±0.88 | 0.50±0.97 | 0.62±1.00 |
| COP | 0.17±0.23 | 0.16±0.40 | 0.17±0.46 |
| CLR | 18.29±26.43 | 14.03±23.08 | 17.38±32.10 |
| CBR | 1.05±1.42 | 0.77±1.36 | 0.98±1.56 |

MLR, monocyte–lymphocyte ratio; NLR, neutrophil–lymphocyte ratio; ELR, eosinophil–lymphocyte ratio; BLR, basophil–lymphocyte ratio; PLR, platelet–lymphocyte ratio; LMR, lymphocyte–monocyte ratio; CAR, CRP-ALB ratio; COP, CRP-preALB ratio; CLR, CRP-lymphocyte ratio; CBR, lymphocyte-BMI ratio.

**Supplemental Table 3. Circulating markers and efficacy in**

**ESCC patients and AGC patients （statistically significant）**

| Markers | Response (ESCC cohort) | | |  | Markers | Disease control (ESCC cohort) | | |
| --- | --- | --- | --- | --- | --- | --- | --- | --- |
|  | PR | Non-PR | *P* value |  |  | Non-PD | PD | *P* value |
| Eosinophil | 0.23±0.11 | 0.12±0.08 | 0.0067 |  | Lymphocyte | 1.62±0.52 | 1.21±0.42 | 0.0420 |
| CD8^+^T | 570.1±239.4 | 352.9±157.4 | 0.0126 |  | ESR | 19.5±17.3 | 38.8±22.8 | 0.0137 |
| CD8^+^CD38^+^T | 215.3±185.6 | 127.8±90.4 | 0.0419 |  | CRP | 13.3±20.7 | 47.4±36.2 | 0.0261 |
| ELR | 0.12±0.05 | 0.08±0.05 | 0.0159 |  | LDH | 176.4±37.7 | 274.6±182.5 | 0.0303 |
|  |  |  |  |  | preALB | 207.6±60.1 | 136.4±49.4 | 0.0039 |
|  |  |  |  |  | ALB | 40.0±3.3 | 35.9±3.76 | 0.0087 |
|  |  |  |  |  | B cells | 132.2±65.3 | 64.6±30.1 | 0.0026 |
|  |  |  |  |  | CAR | 0.36±0.60 | 1.36±1.04 | 0.0171 |
|  |  |  |  |  | COP | 0.10±0.17 | 0.38±0.26 | 0.0195 |
|  |  |  |  |  | CLR | 10.2±19.8 | 41.6±29.4 | 0.0148 |
|  |  |  |  |  | CBR | 0.64±1.05 | 2.11±1.61 | 0.0146 |
| Markers | Response (GAC cohort) | | |  | Markers | Disease control (GAC cohort) | | |
|  | PR | Non-PR | *P* value |  |  | Non-PD | PD | *P* value |
| WBC | 7.9±3.09 | 6.2±2.12 | 0.0320 |  | Eosinophil | 0.18±0.27 | 0.08±0.09 | 0.0284 |
| PLT | 285.5±72.3 | 244.3±103.2 | 0.0413 |  | ELR | 0.14±0.21 | 0.05±0.04 | 0.0109 |
| Neutrophil | 5.88±2.9 | 4.33±1.91 | 0.0270 |  |  |  |  |  |
| Monocyte | 0.46±0.18 | 0.38±0.20 | 0.0215 |  |  |  |  |  |
| CD8^+^CD28^+^T | 192.7±92.6 | 132.1±72.7 | 0.0121 |  |  |  |  |  |

The numbers represent mean±SD.

The statistical significance was performed by Mann-Whitney U test.

**Supplemental Table 4. Circulating markers and efficacy in**

**ESCC patients and AGC patients（no statistically significant）**

| Markers | Response (ESCC cohort) | | |  | Markers | Disease control (ESCC cohort) | | |
| --- | --- | --- | --- | --- | --- | --- | --- | --- |
|  | PR | Non-PR | *P* value |  |  | Non-PD | PD | *P* value |
| WBC | 6.993±0.902 | 7.202±2.086 | 0.7105 |  | WBC | 6.845±1.162 | 8.306±3.296 | 0.5151 |
| Hemoglobin | 135.7±15.42 | 132.2±18.52 | 0.7041 |  | Hemoglobin | 132.6±16.19 | 134.6±23.80 | 0.5091 |
| Platelet | 275.6±68.41 | 240.1±99.22 | 0.1593 |  | Platelet | 256.8±96.19 | 217.4±78.89 | 0.3542 |
| Lymphocyte | 1.787±0.3405 | 1.452±0.549 | 0.0621 |  | Neutrophil | 4.560±1.038 | 6.526±3.407 | 0.1479 |
| Neutrophil | 4.492±0.8068 | 5.124±2.146 | 0.5845 |  | Eosinophil | 0.1607±0.10 | 0.0888±0.06 | 0.0552 |
| Basophil | 0.046±0.0308 | 0.033±0.020 | 0.2755 |  | Basophil | 0.039±0.025 | 0.028±0.018 | 0.3125 |
| Monocyte | 0.4400±0.104 | 0.473±0.181 | 0.7166 |  | Monocyte | 0.4690±0.15 | 0.4500±0.22 | 0.9790 |
| ESR | 13.33±8.246 | 26.76±21.46 | 0.1089 |  | CD4^+^T | 662.5±324.9 | 539.6±185.2 | 0.3454 |
| CRP | 5.770±6.624 | 25.04±30.50 | 0.1135 |  | CD8^+^T | 415.7±209.6 | 362.0±162.1 | 0.5900 |
| LDH | 170.7±37.81 | 205.2±106.2 | 0.1591 |  | CD4^+^/CD8^+^ | 1.879±1.012 | 1.625±0.613 | 0.6102 |
| preALB | 227.2±57.37 | 181.9±63.67 | 0.0621 |  | NK cells | 269.5±203.8 | 230.5±155.6 | 0.7077 |
| ALB | 39.56±2.963 | 39.00±3.973 | 0.7797 |  | Memory CD4^+^T | 487.3±222.3 | 408.0±139.4 | 0.3101 |
| B cells | 144.1±62.89 | 110.0±65.32 | 0.1223 |  | Naive CD4^+^T | 176.1±126.1 | 116.1±73.05 | 0.3539 |
| CD4^+^T | 706.2±193.0 | 615±329.5 | 0.2445 |  | CD4^+^CD28^+^T | 646.9±292.3 | 483.6±155.5 | 0.1620 |
| CD4^+^/CD8^+^ | 1.449±0.6855 | 1.943±0.9870 | 0.1418 |  | CD8^+^CD28^+^T | 203.5±110.4 | 166.5±65.50 | 0.6046 |
| NK cells | 214.3±149.2 | 275.9±205.2 | 0.4203 |  | CD8^+^CD38^+^T | 151.3±125.6 | 137.9±118.5 | 0.5341 |
| Memory CD4^+^T | 541.8±153.3 | 448.6±220.5 | 0.0725 |  | MLR | 0.3217±0.15 | 0.3750±0.18 | 0.3094 |
| Naive CD4^+^T | 158.1±83.15 | 165.1±129.0 | 0.8597 |  | NLR | 3.343±2.087 | 6.399±4.443 | 0.0604 |
| CD4^+^CD28^+^T | 673.2±190.9 | 593.7±297.6 | 0.1535 |  | ELR | 0.0943±0.05 | 0.0725±0.04 | 0.2749 |
| CD8^+^CD28^+^T | 240.8±94.50 | 181.8±103.0 | 0.0877 |  | BLR | 0.0206±0.01 | 0.0238±0.01 | 0.6863 |
| MLR | 0.2533±0.072 | 0.3576±0.168 | 0.1053 |  | PLR | 179.7±103.0 | 195.4±82.72 | 0.4704 |
| NLR | 2.629±0.864 | 4.408±3.250 | 0.1443 |  |  |  |  |  |
| BLR | 0.0278±0.017 | 0.0248±0.013 | 0.8307 |  |  |  |  |  |
| PLR | 160.8±60.62 | 190.0±107.3 | 0.6125 |  |  |  |  |  |
| CAR | 0.1511±0.182 | 0.7017±0.881 | 0.1171 |  |  |  |  |  |
| COP | 0.0333±0.048 | 0.1941±0.242 | 0.0765 |  |  |  |  |  |
| CLR | 3.290±3.757 | 21.03±27.60 | 0.0589 |  |  |  |  |  |
| CBR | 0.2656±0.312 | 1.166±1.433 | 0.1054 |  |  |  |  |  |
| Markers | Response (GAC cohort) | | |  | Markers | Disease control (GAC cohort) | | |
|  | PR | Non-PR | *P* value |  |  | Non-PD | PD | *P* value |
| Hemoglobin | 111.7±27.90 | 114.5±22.35 | 0.7332 |  | WBC | 6.737±2.506 | 7.026±3.051 | 0.9781 |
| Lymphocyte | 1.427±0.569 | 1.296±0.446 | 0.7401 |  | Hemoglobin | 113.5±23.30 | 113.9±28.80 | 0.9999 |
| Eosinophil | 0.1172±0.075 | 0.1844±0.301 | 0.7327 |  | Platelet | 252.0±80.86 | 284.9±146.1 | 0.7298 |
| Basophil | 0.0306±0.016 | 0.0278±0.027 | 0.2017 |  | Lymphocyte | 1.329±0.466 | 1.389±0.605 | 0.7632 |
| ESR | 30.17±26.86 | 21.47±21.11 | 0.2122 |  | Neutrophil | 4.802±2.322 | 5.061±2.716 | 0.9524 |
| CRP | 25.43±49.62 | 14.41±28.29 | 0.4202 |  | Basophil | 0.030±0.025 | 0.023±0.015 | 0.5237 |
| LDH | 214.9±71.90 | 220.2±150.1 | 0.4047 |  | Monocyte | 0.3934±0.15 | 0.4720±0.33 | 0.9694 |
| preALB | 194.6±75.22 | 189.4±62.40 | 0.9746 |  | ESR | 25.61±22.65 | 18.90±26.56 | 0.1416 |
| ALB | 39.61±4.984 | 38.58±4.494 | 0.5997 |  | CRP | 16.52±33.86 | 24.93±48.82 | 0.9187 |
| B cells | 105.4±76.45 | 113.0±62.61 | 0.4418 |  | LDH | 224.0±140.4 | 193.8±48.88 | 0.9694 |
| CD4^+^T | 622.8±234.0 | 501.8±208.3 | 0.0907 |  | preALB | 190.3±65.29 | 195.0±74.03 | 0.8824 |
| CD8^+^T | 425.6±214.8 | 347.5±182.0 | 0.1898 |  | ALB | 39.07±4.668 | 38.30±4.715 | 0.8992 |
| CD4^+^/CD8^+^ | 1.728±0.813 | 1.774±0.949 | 0.9746 |  | B cells | 114.8±72.22 | 91.40±30.81 | 0.4923 |
| NK cells | 286.4±155.8 | 290.9±239.3 | 0.5948 |  | CD4^+^T | 564.6±226.8 | 443.3±180.5 | 0.2100 |
| Memory CD4^+^T | 439.8±222.7 | 359.6±163.4 | 0.1776 |  | CD8^+^T | 382.3±196.4 | 335.0±194.5 | 0.6022 |
| Naive CD4^+^T | 148.6±56.36 | 132.3±113.2 | 0.1688 |  | CD4^+^/CD8^+^ | 1.761±0.829 | 1.748±1.210 | 0.4997 |
| CD4^+^CD28^+^T | 540.9±209.8 | 453.0±187.7 | 0.1837 |  | NK cells | 261.8±159.3 | 410.7±356.6 | 0.3032 |
| CD8^+^CD38^+^T | 175.3±85.78 | 156.0±106.9 | 0.2446 |  | Memory CD4^+^T | 410.7±190.4 | 295.3±128.0 | 0.0648 |
| MLR | 0.3628±0.156 | 0,3211±0.191 | 0.2087 |  | Naive CD4^+^T | 136.2±87.65 | 144.3±139.5 | 0.6021 |
| NLR | 4.662±2.652 | 4.176±3.783 | 0.1660 |  | CD4^+^CD28^+^T | 505.3±200.1 | 381.0±158.4 | 0.0941 |
| ELR | 0.0972±0.080 | 0.1408±0.230 | 0.9964 |  | CD8^+^CD28^+^T | 156.4±82.78 | 133.5±95.85 | 0.2824 |
| BLR | 0.0233±0.011 | 0.0253±0.024 | 0.6049 |  | CD8^+^CD38^+^T | 165.5±101.8 | 149.2±95.25 | 0.6253 |
| PLR | 230.1±104.3 | 211.1±114.1 | 0.3251 |  | MLR | 0.3216±0.14 | 0.3940±0.30 | 0.8135 |
| CAR | 0.7156±1.441 | 0.4203±0.906 | 0.5756 |  | NLR | 4.290±3.505 | 4.547±3.242 | 0.9519 |
| COP | 0.2656±0.635 | 0.1367±0.365 | 0.4436 |  | BLR | 0.025±0.021 | 0.022±0.015 | 0.8585 |
| CLR | 17.64±31.65 | 13.46±25.13 | 0.4979 |  | PLR | 209.7±91.23 | 249.8±170.3 | 0.8139 |
| CBR | 1.038±1.930 | 0.690±1.339 | 0.3842 |  | CAR | 0.4623±0.98 | 0.7670±1.61 | 0.8996 |
|  |  |  |  |  | COP | 0.1593±0.43 | 0.2690±0.65 | 0.8639 |
|  |  |  |  |  | CLR | 13.52±23.02 | 20.74±42.33 | 0.9175 |
|  |  |  |  |  | CBR | 0.7373±1.39 | 1.108±2.21 | 0.9170 |
